# The legacy of past climate warming: strong local adaptation in rear-edge populations

**DOI:** 10.1093/evlett/qrag020

**Published:** 2026-05-07

**Authors:** Antoine Perrier, Olivia J Keenan, Jeremiah W Busch, Laura F Galloway

**Affiliations:** Department of Biology, University of Virginia, Charlottesville, United States; Department of Biology, University of Virginia, Charlottesville, United States; School of Biological Sciences, Washington State University, Pullman, United States; Department of Biology, University of Virginia, Charlottesville, United States

**Keywords:** *Campanula americana*, climate change, genetic diversity, genetic drift, local adaptation, rear edge

## Abstract

Improving forecasts of species’ responses to climate change has become a central challenge in ecology and evolution as species distributions are increasingly disrupted by ongoing climate warming. Insight into this challenge may be gained through a better understanding of evolutionary responses to past climate change. The rear edges of species’ distributions are typically relict populations persisting in former glacial refugia at warmer range limits. As such, they form natural laboratories to study the evolutionary outcomes of past climate warming. We test three hypotheses of possible outcomes to past climate warming at the rear edge of the North American herb *Campanula americana*, including maintenance of high diversity due to long-term persistence, strong genetic drift following habitat decline, and strong local adaptation allowing persistence despite environmental change. Empirical studies rarely explicitly test these hypotheses limiting our understanding of evolutionary responses to warming climates. We tested the three hypotheses, by assessing genetic variation in a genome-wide population genetic study, drift load in a controlled crossing study, and local adaptation in a transplant study. Rear-edge populations exhibited reduced genetic diversity within populations and high differentiation among populations, typically interpreted as evidence of genetic drift, yet showed limited drift load. Instead, these populations expressed strong local adaptation, thriving in rear-edge habitats that were unsuitably warm for populations from the expanded range. Warm-edge populations, therefore, may persist under warming climates by gradually adapting, even in the face of genetic erosion. Our findings highlight the importance of explicitly testing distinct evolutionary trajectories, particularly going beyond measures of genetic variation when inferring evolutionary history. More broadly, these findings identify rear-edge populations not as relics of decline, but as underappreciated models for studying successful adaptation under long-term climate change.

## Introduction

Anticipating global shifts in biodiversity under contemporary climate change (Lenoir & Svenning, 2015; Rubenstein et al., 2023; Urban, 2024) is a central challenge in ecology and evolution. Ecological models generally predict shifts in distribution toward higher latitudes and elevations as organisms track suitable habitats (Parmesan & Yohe, 2003), with risks of extinctions at warmer range limits (Cahill et al., 2014). While these predictions are broadly supported by studies of response to ongoing climate change (e.g., Anderson et al., 2025), there are many exceptions (Alexander et al., 2018; Duchenne et al., 2021; Geppert et al., 2020; Rubenstein et al., 2020, 2023; Vilà-Cabrera et al., 2019). These exceptions highlight the need for a better understanding of how species respond to changing environments. Insights into species’ responses may be gained by studying how evolution in response to past climate change, particularly warming since the Last Glacial Maximum (LGM, ∼20 kya; Hughes et al., 2013), shaped contemporary populations (Davis & Shaw, 2001; de Lafontaine et al., 2018; Hewitt, 2000; Hewitt, 2004).

Rear-edge populations, typically glacial relicts persisting at warmer range limits, can serve as natural laboratories to study evolutionary processes associated with climate change (Hampe & Petit, 2005; Nadeau & Urban, 2019; Perrier et al., 2026a). During the LGM, many temperate species were constrained to glacial refugia at low latitudes and elevations. As the Earth warmed, they expanded their ranges by tracking shifts in suitable habitats (Hewitt, 2000, 2004), with populations at the colonization front forming the “leading edge.” In some species, refugial populations persisted more-or-less in place despite substantial warming and now form the “rear edge” of the species distribution (“stable edge” sensu Hampe & Petit, 2005). The environmental history of these populations offers a unique opportunity to study how warming climates may alter patterns of genetic diversity and structure, affect population demography, and impose new selective pressures. A better understanding of these past evolutionary responses will also enable refining predictions of vulnerability and evolutionary potential under future warming (Perrier et al., 2026a).

Evolutionary responses to past warming can be inferred from genetic patterns in contemporary populations. Theoretical and empirical work on rear-edge populations identify three main outcomes of evolution under past climate change (reviewed in Perrier et al., 2026a). The first is that rear edges are hotspots of genetic variation within and among populations. Rear edges may retain ancestral variation from historic refugial populations, and have greater genetic diversity than populations established during range expansion after the LGM, where serial bottlenecks and founder effects are expected to erode genetic variation (Excoffier et al., 2009). However, only about half of surveyed studies reported the expected greater genetic diversity within rear-edge populations, indicating that simple expectations fail to explain patterns of genetic variation.

The second predicted evolutionary outcome of past climate warming is strong genetic drift in rear-edge populations. Postglacial warming is expected to have caused substantial habitat loss and fragmentation at warmer range limits (Vilà-Cabrera et al., 2019), resulting in long-term demographic decline and isolation of populations. These processes may yield strong genetic drift in rear-edge populations, eroding diversity (Frankham, 1996; Young et al., 1996), reducing adaptive potential (Leonardi et al., 2012; Sánchez-Castro et al., 2022; Willi et al., 2006) and causing fitness decline due to the accumulation of deleterious mutations (i.e., drift load; Kimura et al., 1963; Lynch et al., 1995a; Lynch et al., 1995b; Nei et al., 1975; Kirkpatrick & Jarne, 2000). Rear-edge populations often have low within-population diversity but high between-population differentiation (e.g., Assis et al., 2014; Carbognani et al., 2019; Diekmann & Serrão, 2012), commonly interpreted as a signature of genetic drift (; Perrier et al., 2026a; Pironon et al., 2017). However, this pattern provides only indirect evidence of drift, and does not address whether drift affects fitness and adaptive potential. Indeed, older age and long-term isolation in shrinking suitable habitat may also create patterns of greater differentiation among rear-edge populations than younger populations that resulted from postglacial range expansion (hereafter “expanded range”; Hampe & Petit, 2005, reviewed in Perrier et al., 2026a). We lack studies that explicitly measure drift, e.g., using drift load (but see Perrier et al., 2020; Willi et al., 2018), that would yield conclusive evidence that environmental decline and demographic contraction under past warming has resulted in genetic drift in rear-edge populations.

The third predicted evolutionary outcome of past climate warming at the rear edge is strong local adaptation resulting from long-term directional selection in postglacial climates (Bontrager et al., 2021). This outcome suggests that populations persist not as remnants, destined for decline, but because they have adapted and can exploit the warmer conditions. The few studies that have tested this possibility found heightened genomic signatures of selection (Keller et al., 2018; Parisod & Joost, 2010) and local adaptation (Mathiasen & Premoli, 2016; Saada et al., 2016) in rear-edge populations. Furthermore, a recent meta-analysis found an increase in local adaptation toward equatorial range limits, often home to rear-edge populations (Bontrager et al., 2021), implying that strong local adaptation in rear edges may be more common than currently appreciated. Finally, rear-edge populations often show specializations to warm environments suggesting local adaptation (e.g., Ghouil et al., 2020; Pelletier et al., 2023; Perrier et al., 2025). While many lines of evidence support the persistence of rear-edge populations under climate warming through adaptation, limited direct tests leave this possibility relatively underexplored and potentially underestimated.

Understanding how past climate change has shaped contemporary ecological and evolutionary patterns in rear edges requires evaluating all three outcomes—diversity, drift, and local adaptation. We test these outcomes by integrating climate, genetic, and experimental data on rear-edge populations of the herb *Campanula americana*. We first evaluate whether rear-edge populations are hotspots of genetic variation by assessing patterns of genetic diversity within and among populations (Study 1). We then test for genetic drift by estimating the expression of drift load in a crossing experiment (Study 2). Finally, we estimate local adaptation in a large-scale common garden experiment (Study 3). All studies include populations spanning the range to enable direct comparisons between rear-edge populations and populations in the expanded range. We hypothesize that rear-edge populations will have greater genetic diversity within and among populations, express greater drift load, and/or demonstrate more local adaptation than populations from expanded regions. These complementary approaches provide a holistic framework using rear-edge populations as models to decipher how populations evolve when climates warm.

## Material and methods

### Study system

**Figure 1.**
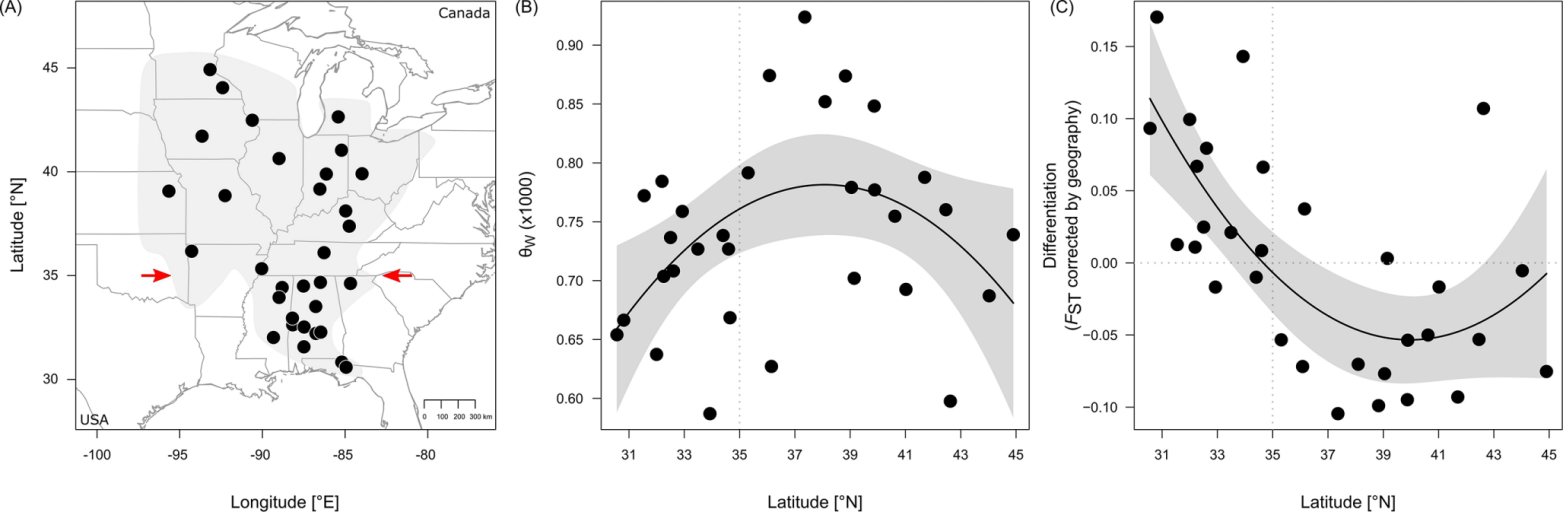
Variation in diversity and differentiation across the range (Study 1). (A) Location of the sequenced populations (dots) used to estimate genetic diversity and differentiation, with the range of the Western lineage indicated (light gray). Arrows represent the latitudinal delimitation of the rear edge. (B) Genetic diversity within populations based on Watterson’s θ_W_ and (C) population-average differentiation among populations (*F*_ST_ after accounting for geographic distance) estimated for each population (dots). In (C), the horizontal dotted line indicates whether differentiation is higher (>0) or lower (<0) than expected based on isolation by distance. Vertical dotted lines represent the separation between the rear edge (<35°N) and the expanded range. Black lines represent the significant model-predicted relationship between each estimate and population latitude, with the 95% confidence interval indicated by shading. Test statistics are reported in Table 1.

*Campanula americana* is a monocarpic herb found in open forested areas across the eastern USA (Figure 1A). Seeds typically germinate in fall (or spring in cold climates) and survive over winter as rosettes. Exposure to cold (i.e., vernalization) is essential to cue reproduction (Perrier et al., 2025), initiated in spring by rosettes elongating into a stalk, i.e., bolting. Plants flower in mid-to-late summer for ca. 5 weeks, and fruit mature ca. 1 month after flowering. *Campanula americana* occupies a large climatic gradient, from subtropical areas close to the Gulf coast in the south to the cold continental Great Lakes region in the north.

The species is divided into three spatially distinct clades (Barnard-Kubow et al., 2015). We focus on largest “Western” clade, as it covers the climatic gradient (Figure 1A). In this clade, the rear edge is geographically defined as the lower latitudinal third of the range (below 35°N; Figure 1A, Perrier et al., 2025). Here, populations overlap with putative glacial refugia and are ancestral in the clade (Barnard-Kubow et al., 2015; Perrier et al., 2026b), indicating a stable rear edge. Within the Western clade, populations in the rear edge can be further divided into three genetic clusters, with one inhabiting the northern region of the rear edge. This clade is basal to postglacial range expansion that includes all populations north of the rear edge (Koski et al., 2019; Perrier et al., 2026b; Prior et al., 2020). Contemporary rear-edge habitats are less suitable than the expanded range (Barnard-Kubow et al., 2015; Perrier et al., 2026b) and are substantially warmer, especially in winter, with populations expressing reduced vernalization requirements and a distinctive delay in flowering (Perrier et al., 2025).

### Study 1: Are rear edges reservoirs of diversity?

We assessed the hypothesis that rear-edge populations are hotspots of genetic variation by testing for differences in genetic diversity within populations and in genetic differentiation among populations across the species’ range. We include both central and northern leading-edge populations to fully represent expansion history as this hypothesis is predicated on genetic variation declining with range expansion.

### Sequencing and genotyping

We sequenced 31 populations spanning the distribution of *C. americana*’s Western clade (Figure 1A, Table S1). We collected leaf tissue from 7 to 10 individuals per population (292 total, Method S1). DNA extraction was performed by the Genomics & Cell Characterization Core Facility at the University of Oregon (Eugene, OR, USA). Sequencing was performed using a Restriction-site Associated DNA sequencing (RAD-Seq) approach (Andrews et al., 2016; Baird et al., 2008) by Floragenex Inc. (Beaverton, OR). Raw reads were processed into single nuclear polymorphism (SNP) calls using a modified STACKS v2 pipeline (Rochette et al., 2019), then filtered to retain a final set of 137,312 high quality SNPs. As this species is an old autotetraploid (Lopez-Caamal et al., unpublished data), diploid genotypes were converted into tetraploid genotypes using a custom R script. Details on plant material, DNA extraction, library preparation, sequencing, SNP call, and processing, are provided in Method S1.

### Estimates of genetic variation

We estimated population-level genetic diversity as Watterson’s (θ_W_) and Tajima’s (θ_π_) estimators of θ (Tajima, 1989; Watterson, 1975). Both are measures of diversity, but θ_W_ is more sensitive to rare variants (segregating sites) than θ_π_, which measures average pairwise differences. These estimates were calculated for each population in a sliding window approach using a custom R script (adapted from Koski et al., 2019; Method S1), then averaged at the level of the population. We estimated differentiation among populations by first calculating pairwise F_ST_ (Weir & Cockerham, 1984) using the *StAMPP* package in R (Pembleton et al., 2013). To account for isolation-by-distance (IBD; ; Slatkin, 1993), we tested the relationship between linearized pairwise *F*_ST_ estimates (Rousset, 1997) and pairwise geographic distances, using the residuals from this relationship to quantify population-level genetic differentiation relative to IBD expectations (hereafter “differentiation,” Methods S1).

To evaluate the expectation that rear edges are hotspots of genetic diversity, we assessed whether population-level estimates of θ_W_ and θ_π_ were explained by a population’s latitude using linear models in R (R Core Team, 2025). For all analyses including latitude, we initially evaluated whether latitude was best described by a linear or a second-degree quadratic relationship (c.f. Pironon et al., 2017), and results are reported for the best model based on corrected Akaike Information Criterion scores (AICc, Sugiura, 1978). If both models performed equally well (|ΔAICc| ≤ 2), the model with highest *R*^2^ was chosen. Dependent variables were tested assuming a normal distribution. We tested and confirmed assumptions of each model.

### Study 2: Do rear-edge populations suffer from strong genetic drift?

We tested the hypothesis that rear-edge populations show elevated drift load, consistent with a history of strong genetic drift, by quantifying heterosis across the range in a crossing experiment. Heterosis, the increased fitness of between-population crosses (BPC) relative to within-population crosses (WPC), reflects the masking of recessive deleterious mutations presumably accumulated under drift in the parental populations (Crow, 1987).

### Crossing design, raising of offspring, and recording of traits

**Figure 2.**
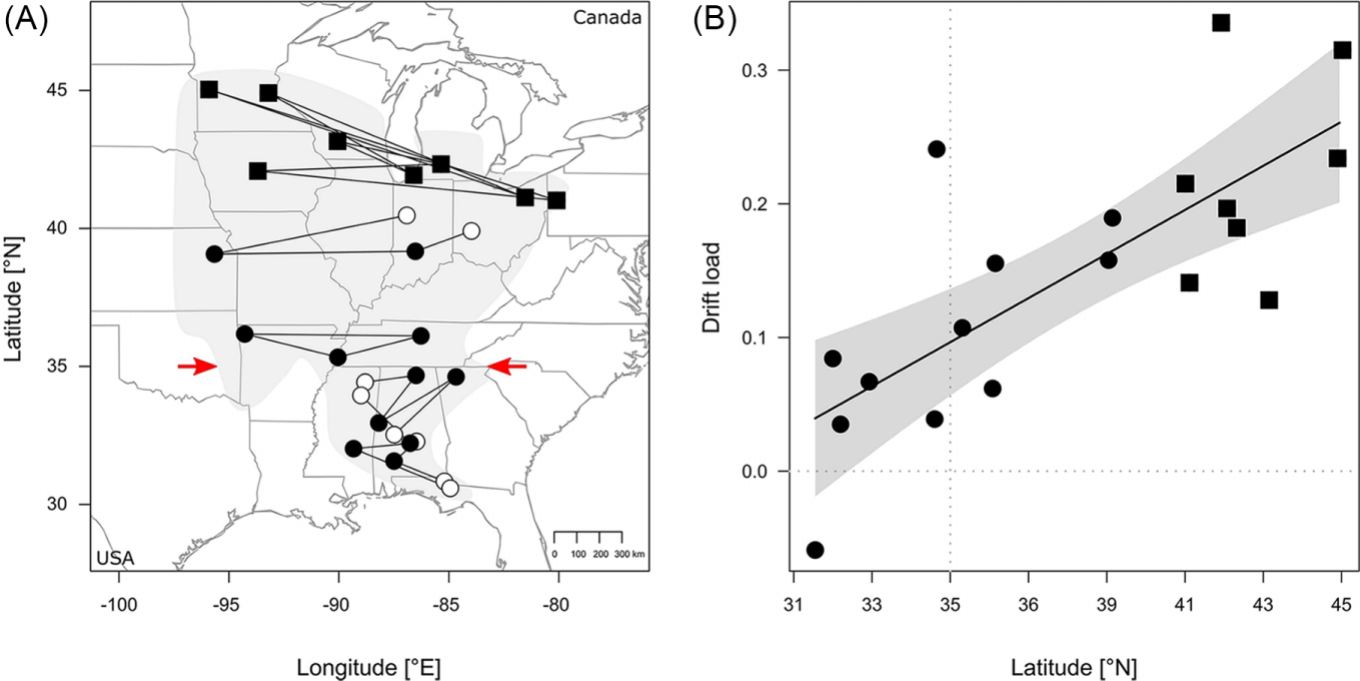
Variation in the expression of drift load across the range (Study 2). (A) Location of the populations (dots) crossed (lines) to estimate drift load, with focal populations in black and partner populations in white. Black squares represent additional populations from Koski et al. (2019). The range of the Western lineage is shaded light gray, with arrows representing the latitudinal delimitation of the rear edge. (B) Drift load of each focal population (dots) and additional populations (squares) calculated from heterosis estimates of BPC. Values above 0 (horizontal dotted line) indicate drift load expression, values below 0 represent outbreeding depression. The vertical dotted line represents the separation between the rear edge (<35°N) and the expanded range. The solid line represents the significant relationship between drift load and latitude, with the 95% CI indicated in gray shading. Test statistics are reported in Table 1.

The experimental design was similar to a previous study investigating the accumulation of drift load in the expanded range of the species (Koski et al., 2019) to allow for comparison. We selected 19 populations spanning the rear edge and the range center (Figure 2A, Table S2A). Maternal seed families were collected in nature, and for each population we grew one seedling from up to 18 families (mean 14.5 families). Seedlings were raised in controlled environments that simulated the species’ seasonal cycle with germination and initial growth in growth chambers simulating fall, followed by simulated winter in a cold room, then reproduction in a greenhouse with conditions simulating summer (Method S2).

For each population, we performed crosses among unrelated individuals (WPCs). We also crossed between populations (BPCs) from similar latitudes (Figure 2A), minimizing environmental differences and creating 15 population-pairs. Of the 19 populations, 11 served as pollen recipients (mother) in one pair and as pollen donors (father) in another pair, while the remaining 8 contributed to a single pair, serving as only a pollen donor or a pollen recipient (Table S2B). Crosses generated 19 WPC lines and 15 BPC lines, with an average 13 seed families per line (i.e., maternal–paternal plant combinations; Table S2B, Method S2).

To estimate heterosis, we raised on average 21 seedlings for each line (Table S2) using similar procedures as described above (Method S2), resulting in a total of 428 WPC and 296 BPC plants. Over the course of the experiment, we tracked germination, survival, whether plants flowered, and estimated reproductive output from flower counts (Method S2).

### Estimation of drift load

We first calculated lifetime fitness at the level of the pot as the product of germination proportion and reproductive output, set to 0 if the plant died or did not flower. Lifetime fitness was averaged across replicates of a seed family within each WPC or BPC line, and then at the level of the line resulting in a single mean for each WPC and BPC line (Table S2). Heterosis (H) was calculated for each BPC line as the lifetime fitness *(W)* of the BPC line compared to the average lifetime fitness (*W̄*) of the WPC of its two parental populations standardized by the average of the parental populations: *H* = (*W*_BPC_—*W̄*_WPC_)/*W̄*_WPC_ (Table S2B). Population-level drift load was then estimated for each of the 11 populations that participated in two BPC crosses as the average heterosis across the two BPC lines (Table S2A). We supplemented this data with drift load estimates for eight northern leading-edge populations from Koski et al. (2019; Table S2A) to capture the range of drift load across the species.

Using this combined dataset, we assessed whether rear-edge populations express higher drift load than populations in the expanded range by testing the effect of a population’s latitude on drift load in a linear model using R, assuming a normal distribution. High latitude BPCs were on average further apart from one another than those at low latitudes, potentially affecting patterns of drift load due to greater IBD and thus differentiation. We therefore explored whether population-average linearized *F*_ST_ (Rousset, 1997) is associated with estimates of drift load in a linear model. Population-average *F*_ST_ estimates from Koski et al. (2022) from a similar RAD-Seq dataset were used for the eight high-latitude populations.

### Study 3: Do rear-edge populations show strong local adaptation?

We tested the hypothesis that rear-edge populations exhibit stronger adaptation to local climates than the rest of the range due to a long history of selection under past warming using a large common garden experiment. This experiment included sites representing rear-edge and range-center environments, and tested for local adaptation as a fitness advantage of populations growing in environments similar to their home climate (i.e., “local vs foreign” criteria; Kawecki & Ebert, 2004).

### Experimental design and recording of traits

**Figure 3.**
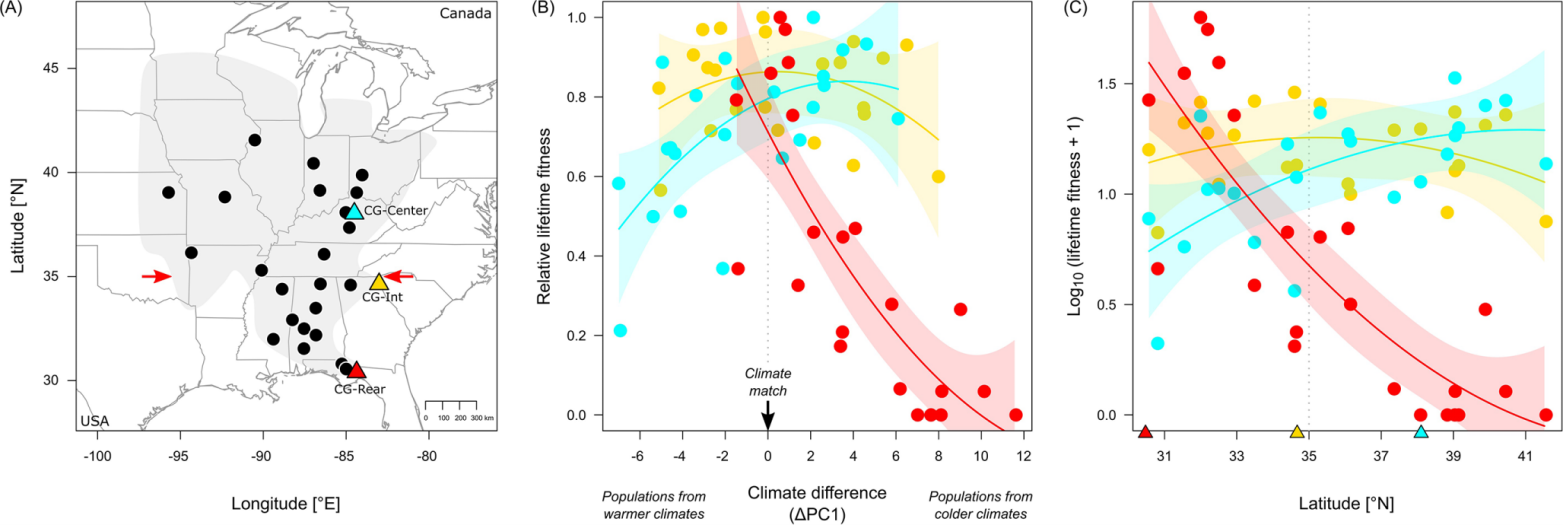
Variation in local adaptation across the range (Study 3). (A) Sampling of populations (dots) and common gardens (triangles), with the range of the Western lineage shaded. Arrows represent the latitudinal delimitation of the rear edge. Relative lifetime fitness (B) and average lifetime fitness (C) of populations (dots) raised in each common garden (distinguished by color as in A). Population averages were calculated from log10 transformed family averages (after adding + 1 so that all values are positive). Solid lines represent the relationship between fitness and the climate difference between each garden and that of the population’s location of origin (B, ΔPC1), and population’s latitude of origin (C, latitude of gardens indicated by triangles); 95% CI indicated in color-matching shading (test statistics: Table 2, Tables S7 and S8). The dashed line indicates a climate match between the population’s origin and the garden (climate difference = 0, B) or the separation between the rear edge (<35°N) and the expanded range (C).

We selected 23 central and rear-edge populations (Figure 3A, Table S3) to be raised in three common gardens (CG, Figure 3A, Method S3). Gardens were located along an 850 km latitudinal gradient, representing conditions in the center of the range (CG-Center, Lexington KY, 38.1°N), the rear edge (CG-Rear, Tallahassee FL, 30.5°N), as well as intermediate between the two (CG-Int, Clemson SC, 34.7°N). This experiment was also used to assess variation in phenology (Perrier et al., 2025).

Plants from each of the 23 populations were raised in two cohorts in each common garden (Method S3). The “winter cohort” captured how winter conditions affect survival and the cuing of reproduction, while the “summer cohort” captured the effect of spring and summer conditions on survival and reproduction of seedlings adequately cued for reproduction. Use of adequately cued plants yielded a predictable sample size for reproductive traits but came at a cost of observing potential delays in flowering time associated with shortened vernalization (see Perrier et al., 2025). For each cohort and garden, we raised 30 seedlings per population (2 individuals/family × 15 families/population) for a total of 4,140 individuals (Table S3).

Plants for the summer cohort were germinated and vernalized in controlled conditions over the winter, transplanted in the field in spring, and harvested in late summer when most had mature fruits (Method S3). Seeds for the winter cohort were germinated in controlled conditions and rosettes transplanted in each garden before the onset of winter (Method S3). Data collection finished late spring, allowing time for all plants (that were successfully cued) to bolt after winter. Garden locations were selected to mimic natural habitats of the species (Method S3; Perrier et al., 2025). For each cohort and garden, air temperature was monitored hourly with a data logger (iButton®; Maxim Integrated Products Inc., San Jose, CA, USA) placed 1.5 m aboveground in the shade.

We recorded traits across the life cycle (minus germination) for each individual by visiting the gardens multiple times in summer for the summer cohort, and in spring and early summer for the winter cohort (Method S3). At each visit, we assessed survival and transition to bolting, and for the summer cohort, transition to flowering (i.e., production of at least one bud). Mature summer-cohort plants were collected to record reproductive output (total number of fruits, flowers, and buds; Method S3).

### Climate difference between common gardens and populations

We quantified climate differences between the conditions experienced in the common gardens and each population’s location of origin with 14 seasonal temperature and precipitation variables and 5 bioclimate variables (Table S4, Method S3). The seasonal variables included averages of daily mean, minimum and maximum temperature, and the sum of daily precipitation for the winter, spring, and summer growing season as well as number of days of frost and of vernalization. The bioclimate variables were selected to reflect temperature and precipitation seasonality (Method S3). For gardens, temperature and precipitation data came from dataloggers and nearby weather stations during the experiment; for populations, from the PRISM database (2018–2022; https://prism.oregonstate.edu, date accessed May 13, 2024).

We performed a Principal component analysis (PCA) on the population climate variables to reduce dimensionality and autocorrelation. Climate variation was mostly explained by an axis summarizing variation in winter and spring temperature (PC1, 76.65% of variance explained; Table S5), and by an axis summarizing variation in precipitation and summer temperatures (PC2, 8.81% of variance explained; Table S5). The gardens were projected into this PC space and captured the range of variation along PC1 but less variation along PC2 (Figure S1). We thus focused on PC1 to assess local adaptation. For each population in each garden, we calculated the climate difference (ΔPC1) between the garden’s PC1 and the population’s home PC1 (ΔPC1 = PC1_garden−_PC1_population_), where a value of 0 indicates climate matching, i.e., the population’s climate is “local” to the garden, while positive or negative values indicate that populations experience warmer or colder conditions in the garden than at their location of origin. To verify that our 23 populations yielded a PC1 that was representative of the overall distribution of the clade, we conducted the same analyses on a sample of 238 populations derived from *iNaturalist* observations (Perrier et al., 2025) and found very similar results (analysis not shown).

### Assessment of local adaptation

We generated a measure of lifetime fitness across the two cohorts by combining five traits summarizing fitness at key life stages: survival over winter (binary), survival until reproduction (binary), bolting (binary), flowering (binary), and reproductive output (continuous, Method S3). Traits were averaged at the seed family level in each garden. For a few family–garden combinations, data on traits in either cohort was missing (summer cohort: 20 of 992, winter cohort 43 of 1,015). For these cases, missing trait data was imputed as the average across populations in the garden. Family averages were then multiplied across life stages to estimate lifetime fitness. Fitness was then averaged for each population within each garden after log10 transformation (+1 so that all values are > 0) to achieve normality (Table S3). Population-level lifetime fitness was standardized to account for variation in garden quality by dividing each value by the maximum value within that garden.

To evaluate whether local adaptation is stronger at the rear edge, we tested how variance in fitness depended on the climate difference between the population’s home and the gardens. If populations are locally adapted, we expect fitness in a given garden to be highest for populations whose home climate matches the garden’s climate (ΔPC1 = 0), and to decline as the climate difference increases (local vs foreign; Kawecki & Ebert, 2004). Stronger declines in fitness indicate stronger local adaptation. Variation in relative lifetime fitness was tested in a hierarchical mixed effect model using the R packages *lme4* (Bates et al., 2015) and *blme* (Dorie et al., 2026), assuming a normal distribution. Fixed effects included common garden (three levels), climate difference (ΔPC1), and their interaction (Method S3). Population was included as a random effect. The effect of climate difference was modeled using a quadratic relationship to allow fitness to decline towards positive and negative values of ΔPC1. We used post hoc tests (Lenth, 2019) to compare the fitness effects of climate difference among gardens. Finally, we tested the extent to which variation in lifetime fitness was explained by a population’s latitude (fixed effect) and whether this relationship varied among gardens.

## Results

### Study 1: Are rear edges reservoirs of diversity?

Rear-edge populations are less genetically diverse but more genetically differentiated than central populations. Patterns of genetic diversity within populations and differentiation among populations were best explained by a quadratic relationship with latitude (Table S6). Diversity based on θ_W_ was highest for mid-latitude populations and significantly declined at high and low latitudes (Table 1, Figure 1B). While not significant, θ_π_ followed a similar pattern (Table 1, Figure S2). In contrast, genetic differentiation between populations, accounting for geographic distance, was lowest at midlatitudes and increased substantially toward low latitudes, with the most southern populations having three times greater differentiation. Genetic differentiation also increased toward high latitudes though the change was relatively modest (Table 1, Figure 1C).

**Table 1.** Test of variation in genetic diversity (ϴ_π_ and ϴ_W_) and differentiation (Study 1), and drift load (Study 2), across the range of *C. americana*.

Dependent variable		Latitude			
	*N*	*β*_linear_	*β*_square_	*F*	*R* ^2^
ϴ_W_	31	**8.05e-05**	**−1.82e-04**	3.46*	0.14
ϴ_π_	31	−2.98e-05	−8.93e-05	1.97	0.06
Differentiation	31	**−2.35e-01**	**1.59e-01**	12.65***	0.44
Drift load	20	**1.65e-02**	−	22.37***	0.54

Dependent variables were assumed to follow Gaussian distributions. Test statistics include the slope of the effect of latitude (*β*) estimated for the linear term and if applicable the squared term (Table S6), the *F* value and the *R*^2^ of each model. Values are in bold if *p*-values < .05; * *p* < .05; and *** *p* < .001.

### Study 2: Do rear-edge populations suffer from strong genetic drift?

Rear-edge populations expressed less drift load than the expanded range. Most populations across the range displayed some load (Figure 2B), with between-populations crosses outperforming within-populations crosses to yield positive values of drift load. Variation in drift load was best explained using a linear model (Table S6), and load increased almost fivefold with latitude (Table 1, Figure 2B). There was no relationship between drift load and linearized F*_ST_* (*F* = 0.01, *p* = .93, *R*^2^ = 0.00, Figure S3), indicating IBD did not drive the increased drift load at higher latitudes.

### Study 3: Do rear-edge populations show strong local adaptation?

Local adaptation, while common across the range, is highest at the rear edge. In each garden, populations from climates more similar to the garden (i.e., climate-matching) generally had higher fitness than populations from warmer or colder climates (ΔPC1 Table 2, Table S7A, Figure 3B). The strength of local adaptation, indicated by the slope of fitness change with climate difference (Figure 3B), was similar in the central and intermediate gardens, but stronger in the rear-edge garden (CG * ΔPC1 Table 2, Table S7B). In the rear-edge garden, populations from increasingly colder climates had increasingly lower fitness, with most northern populations having a fitness of zero (Figure 3B).

**Table 2.** Test of variation in local adaptation (Study 3) across the range of *C. americana*.

Fixed effects	*Χ* ^2^
Common garden (CG)	6.02*
Climate difference (ΔPC1)	10.75**
CG * ΔPC1	18.17**

The dependent variable, relative population-average lifetime fitness, was assumed to follow a Gaussian distribution. All fixed effects were tested in one model, optimized with the *bobyqa* optimizer to improve convergence. Test statistics include the chi-squared value of the model, bold if *p*-values < .05; * *p* < .05; and ** *p* < .01. Marginal *R*^2^*_m_*= 0.70 (fixed effect only); *R*^2^*_c_*= 0.83 (fixed and random effects). The slope of each effect and post hoc tests are reported in Table S7. Results for random effects are not shown.

The analysis of lifetime fitness across latitudes yielded similar results (Table S8). Fitness in each garden was highest for populations originating from similar latitudes as the garden (Figure 3C), with the highest fitness overall in low-latitude rear-edge populations in the rear-edge garden.

## Discussion

### Rear edges are neither reservoirs of genetic diversity nor suffer from drift load

Rear-edge populations, relicts that persist in glacial refugia, offer unique insight into how populations evolve under long-term climate change. In *C. americana*, past climate warming left mixed signatures of evolutionary processes at the rear edge. Estimates of genetic variation do not support the hypothesis that these populations act as reservoirs of genetic diversity, as expected for stable rear edges (Perrier et al., 2026a). Instead, they point toward strong genetic drift with lower within-population diversity at the rear edge than in central populations, particularly for segregating alleles (θ_W_). Rear-edge populations also showed markedly higher differentiation among populations compared to the rest of the range, including the leading edge. Diversity between populations rather than within them is commonly observed in rear edges, and often attributed to strong genetic drift (e.g., Assis et al., 2014; Carbognani et al., 2019; Diekmann & Serrão, 2012; but see Wood et al., 2021).

Long-term climate change provides context for these patterns. Rear-edge habitats served as refugia (Barnard-Kubow et al., 2015; Perrier et al., 2026b) and were probably highly suitable for *C. americana* during the LGM. However, their suitability is now low (Barnard-Kubow et al., 2015; Perrier et al., 2026b), likely reflecting a long history of habitat degradation from postglacial warming. Habitat degradation is expected to lead to demographic decline, progressive isolation, and a reduction in diversity in populations. Within-population diversity also declined toward the leading edge, likely reflecting genetic erosion of populations established through repeated bottlenecks during postglacial range expansion (Excoffier et al., 2009). Together, these patterns suggest that past climate warming led to reduced diversity at both range edges, likely through different mechanisms, with expansion-driven erosion at the leading edge and long-term habitat decline at the rear edge (see also ; Hampe & Petit, 2005; Pironon et al., 2017).

Contrary to expectations, rear-edge populations expressed the lowest drift load across *C. americana*’s range. Therefore, results do not support the hypothesis that these populations have experienced strong genetic drift. Further, the results contradict expectations of drift inferred from estimates of genetic variation and habitat decline at the rear edge (Barnard-Kubow et al., 2015; Perrier et al., 2026b). These results also contrast with the only previous evaluation of drift load at the rear edge that found it elevated and of similar magnitude to the leading edge in *Arabidopsis lyrata* (Perrier et al., 2020; Willi et al., 2018). Instead, we found drift load only increased towards the leading edge, consistent with the expectation that drift increases with range expansion (Peischl et al., 2013, 2015). While past warming appears to have led to an expansion-driven history of drift at the leading edge, other evolutionary processes—beyond genetic drift—appear to have shaped genetic diversity and differentiation at the rear edge.

### Strong local adaptation allowed rear-edge populations to persist in changing habitats

In the common garden experiment, populations generally performed better in climates similar to conditions at their location of origin, consistent with local adaptation (Kawecki & Ebert, 2004; Leimu & Fischer, 2008). This pattern was strongest in the rear-edge garden, where fitness declined sharply as the climate became increasingly different from a population’s home. Strong local adaptation at the rear edge likely reflects a history of persistent directional selection under ∼20 kya of postglacial warming, confirming the third hypothesis. This long-term environmental change likely provided the selective pressure and time required for gradual adaptation to increasingly warm conditions, enabling rear-edge populations to persist in place despite experiencing dramatic habitat changes. Local adaptation was weaker further north, where populations tracked shifts in suitable habitats and therefore experienced less environmental change, providing less opportunity for local adaptation. Elevated local adaptation toward the equatorial range limit is common across temperate species (Bontrager et al., 2021). Our findings experimentally corroborate this pattern and demonstrate strong local adaptation as a potential hallmark of rear edges.

Rear-edge populations in *C. americana* thrived in their local garden despite environmental conditions that appear marginal for the species. In the rear-edge garden, populations from the expanded range had very low fitness, suggesting contemporary rear-edge habitats fall outside of the climate niche occupied by most of the species. Low fitness was mainly due to a lack of bolting (Perrier & Galloway, 2026). The brief 2 days of vernalization experienced in this garden (Table S4), typical of the subtropical rear-edge climate (Perrier et al., 2025), was likely insufficient to cue reproduction in populations sourced from colder climates. Our findings of low habitat suitability at the rear edge of the species is consistent with results from niche modeling (Barnard-Kubow et al., 2015; Perrier et al., 2026b), and reflects general expectations of the marginality of rear-edge habitats (Bontrager et al., 2021; Vilà-Cabrera et al., 2019). Despite this, plants from rear-edge populations did not suffer from low-quality habitat in the rear-edge garden, and in fact achieved some of the highest performance across populations and gardens. Our findings contribute to growing evidence that rear-edge populations may harbor unique adaptations to warming (Ghouil et al., 2020; Pelletier et al., 2023; Perrier et al., 2025), allowing them to thrive in habitats that are unsuitable relative to the species’ overall niche. These findings echo recent critiques of predicted marginality at the rear edge (Vilà-Cabrera et al., 2019), that often do not take local adaptation into account. As we demonstrate here, overlooking local adaptation may skew expectations of performance at range limits.

### Revisiting expectations of evolution under warming climates

Our study in *C. americana* offers new insight into how past climate change may have shaped the evolution of rear-edge populations. Most studies on the rear edge evaluate one of two possible evolutionary trajectories—high levels of ancestral diversity or drift-driven decline (Perrier et al., 2026a). We demonstrate a third, often overlooked, possibility that rear-edge populations can persist and even thrive under long-term climate change, not because they maintain high genetic diversity, but because they have gradually adapted to changing conditions. This third evolutionary trajectory offers a more nuanced framework for understanding response to past climate change, and spotlights rear edges as models of successful adaptation to continuously warming climates.

Our study also challenges the assumption that reduced diversity and heightened differentiation are reliable evidence for strong genetic drift. This pattern has been found in many studies, contributing to the prevalent notion that rear edges experience strong drift (reviewed in Perrier et al., 2026a). Yet, our results indicate that this assumption may not be accurate and suggest other evolutionary forces may create this pattern. For example, selection linked to strong local adaptation could have driven selective sweeps reducing diversity (De La Torre et al., 2021; Wei et al., 2023). Likewise, high differentiation of populations at the rear edge might be the result of the long history of populations in this area. Only a few studies have gone beyond estimates of genetic variation to test for drift using genetic signatures of population decline (Cho et al., 2020; Dupoué et al., 2021; Kvist et al., 2015; Lepais et al., 2022; Rodríguez-Muñoz et al., 2007), or drift load (Perrier et al., 2020, 2022). One of those concluded low diversity and high differentiation in seaweed may result from past selection, not drift (Wood et al., 2021). The lack of explicit testing, and the fact that local adaptation can hide in plain sight masquerading as drift, raises the question of how often rear-edge populations thought to have been molded by drift are actually shaped by historic selection and adaptation? Resolving this question will require broader consideration and evaluation of evolutionary history at the rear edge.

### Evolutionary legacies and the fate of rear-edge populations under future climates

Past evolutionary processes may influence how rear-edge populations respond to future climate change. For example, rear-edge populations are often expected to experience high drift (Hampe & Petit, 2005; Perrier et al., 2026a), raising widespread concerns about their long-term persistence (Nadeau & Urban, 2019; Vilà-Cabrera et al., 2019). In small range-edge populations, drift can indeed reduce adaptive potential, reduce fitness, and increase vulnerability to warming stress (Koski et al., 2019; Perrier et al., 2020, 2022; Sánchez-Castro et al., 2022). As we show here, this vulnerability at the rear edge may be overestimated, because drift is often inferred from estimates of genetic variation alone. Similarly, strong local adaptation at the rear edge suggests that rear-edge populations may be less vulnerable to climate warming than predictions based on environmental suitability of the species as a whole. For example, in *C. americana*, rear-edge populations are expected to be more resilient to future warmer winters than more northern populations due to reduced vernalization requirements that evolved in response to past warming (Perrier et al., 2025, 2026b). Although, specialization to current local climates may lead to increased risk of maladaptation under future climates (Anderson & Wadgymar, 2020; Frank et al., 2017; Kooyers et al., 2019). In sum, our study highlights the importance of including a robust assessment of the evolutionary legacy of past warming in projections of responses to future warming.

## Conclusion

Our study in *C. americana* highlights rear-edge populations as powerful models to understand how populations evolve under long-term climate change. Realizing this potential requires the integration of genomic and experimental approaches as well as the consideration of multiple possible evolutionary trajectories. More broadly, our study suggests that rear-edge populations are not necessarily relics in decline but products of successful, gradual adaptation to warming climates. As such, they represent valuable yet underappreciated natural laboratories to study the mechanisms enabling persistence under changing climates. Past evolutionary processes may also influence how rear-edge populations respond to future climate change. Recognizing successful adaptation—not just vulnerability—as a possible response to past warming could provide valuable insight for forecasting and managing species responses to future warming.

## Supplementary Material

qrag020_Supplemental_File

## Data Availability

Raw read data are publicly available in the Sequence Read Archive (BioProject PRJNA1306192). Custom scripts necessary to recapitulate our genetic analyses, as well as the diploid VCF, loci-level summary statistics, pairwise *F*_ST_ and geographic distance, fitness data of the drift load, and local adaptation studies, are stored on Zenodo (https://doi.org/10.5281/zenodo.16883804).
