# Supplementary material for: The legacy of past climate warming: strong local adaptation in rear-edge populations
Source: Evol Lett. 2026 May 7;10(4):343–54. doi: 10.1093/evlett/qrag020 (PMC13451775; doi:10.1093/evlett/qrag020)
Supplement: Supplementary file 1 [file qrag020_supplemental_file.pdf]

Supplementary Methods

Method S1: Population genetic study (Study 1)

Plant material

We collected leaf material from seven to ten individuals of 31 populations, for a total of 292 individuals (Fig. 1, Table S1.1). Leaves were collected on individuals raised in the greenhouse from seeds harvested from presumably unrelated maternal plants in natural populations (107 samples) or from greenhouse produced seeds (independent within-population crosses, 185 samples, see Method S2). Samples were gently dried in an oven at low heat (~35 to 40 °C) for two to three days, then preserved at -20 °C until sequencing.

DNA extraction, library preparation & sequencing

Samples were sequenced using a Restriction-site Associated DNA sequencing approach (RAD-Seq; Andrews et al., 2016; Baird et al., 2008). These samples were part of a larger sequencing project of 475 individuals (Bioproject PRJNA1306192). DNA extraction was done by the Genomics & Cell Characterization Core Facility (GC3F) of the University of Oregon (Eugene, OR, USA). For each sample, DNA was extracted from at least 20mg of dry leaf tissue (MagMAX™ Plant DNA Isolation Kit, Applied Biosystems, Foster City, CA, USA) then purified (Mag-Bind® TotalPure NGS, Omega Bio-tek, Norcross, GA, USA). Samples were then concentrated, tested for purity and fragmentation, and equilibrated to 20 ng/µL.

Library preparation and sequencing was performed by Floragenex, INC (Beaverton, OR). Samples were processed in five batches of 95 individuals from extraction to sequencing. Individual samples were digested with the *PstI* restriction endonuclease, and resulting fragments were ligated with adaptors containing unique 10 bp barcodes to identify each sample. Fragments were pooled by groups of 95 (multiplexed), sheared by sonication, size selected (300–500 bp range) and amplified by PCR. Libraries were paired-end sequenced (150bp) on two S4 flow cell lanes per library using a NovaSeq6000 (Illumina, Inc. San Diego, CA, USA). For two of the five libraries, initial sequencing had low yields. These libraries were sequenced two additional times.

Processing of raw reads

Raw reads were processed using a modified STACKS v2 pipeline for RAD-Seq data using a reference genome (Rochette et al., 2019). Multiplexed reads were first quality-checked using *fastqc* (Andrews, 2010). For each sequencing lane and library, paired reads were demultiplexed (assembled by individual), filtered for low quality and missing bases (-c -q) and trimmed to remove barcodes using *process-radtags* in STACKS. For each individual within each lane, PCR duplicates were removed using *clone_filter* in STACKS. The remaining sequence data was then merged within individuals across sequencing lanes. For each individual, forward and matching paired-end reads were aligned to a *C. americana* reference genome (haploid chromosome-level assembly based on the Appalachian clade population VA73 using PacBio HiFi reads; Lopez-Caamal et al.,, *unpublished data*) using BWA-MEM (Li, 2013; Li & Durbin, 2009). Based on these alignments, we generated a catalog of RAD loci (groups of homologous reads derived from the same genomic region and assembled across samples) including all individuals, using *gstacks* in STACKS. In total, the catalog comprised 1,566,971 RAD loci, assembled from 924,427,374 forward reads and 748,416,814 matched paired-end reads, for an average coverage of 31.2x per individual (SD = 9.7x; range: 8.8x – 80.6x). RAD loci averaged 465bp in length with a mean insert length of 72bp, for a total length of 615 Mbp, covering about ~36% of the 1.7 Gbp haploid reference genome (Lopez-Caamal et al.,, *unpublished data*).

Genotyping

Genotypes were called from the catalog generated above as variant call format (VCF) using *populations* in STACKS. We retained SNPs where sequence information was present for at least half of the individuals within each population (-r 0.5, not taking account populations where the site was entirely missing) and for 85% of individuals overall (-R 0.85), and with a minor allele count of at least 2 (--min-mac 2) to include rare variants for accurately calculating diversity estimates while removing singletons that could result from errors during sequencing. The resulting 158,356 SNPs were filtered to exclude 16,778 SNPs with a mean read depths of less than 20 (following Koski et al., 2019) or greater than 67 (average coverage + 4*√average coverage, following (Li, 2014); average before filtering = 41) using VCFTools (Danecek et al., 2011). In addition, 4,266 variants resulting from indels were excluded using BCFTools (Danecek et al., 2021). This resulted in a final set of 137,312 high quality SNPs (average coverage of 38.9x reads

per individual) distributed across 7,602 RAD loci, which together span ~7.3 Mbp, corresponding to ~0.4% of the genome sequenced in each individual. For each sample and at each SNP, diploid genotypes were converted into tetraploid genotypes to account for partial heterozygotes. Tetraploid genotypes were estimated using a custom R script by first calculating the ratio of reads supporting the derived allele (B) over the reference allele (A) from the number of reads provided in the VCF file, then assigning genotypes using following bins: ≤ 0.05, AAAA; <0.375, AAAB; <0.625, AABB; <0.95, ABBB; ≥ 0.95, BBBB. This method was used in a previous study in the species to estimate tetraploid genotypes from RAD-Seq data (Prior et al., 2020), with the difference that we added the thresholds of 0.05 and 0.95 to bin homozygous genotypes (instead of 0 = AAAA and 1 = BBBB), to account for potential sequencing errors. These thresholds represent cases where out of the minimum of twenty reads necessary to retain a SNP, only one read supports the minor allele.

Accounting for isolation by distance in measures of genetic differentiation among populations

Because sampling across the range was not homogeneous and genetic differentiation among populations is often expected to increase with geographic distance due to isolation-by-distance (IBD; Slatkin 1993; Eckert et al., 2008), we accounted for IBD before assessing variation in differentiation across the range. We estimated differentiation by calculating pairwise F_{ST} (Weir & Cockerham, 1984) among populations using the *StAMPP* package (Pembleton et al., 2013) in R (R Core Team, 2025). We also estimated geodesic geographic distance (km) for each pair of populations using the harvestine method implemented in the *geodist* package in R (Karney, 2013). Pairwise F_{ST} estimates were linearized (F_{ST} / (1- F_{ST}) to account for the non-linear relationship between F_{ST} and geographic distance under isolation-by-distance models (Rousset, 1997), and included in a linear model testing for the effect of pairwise geographic distances (R^2= 0.08, P < 2e-16). Residuals of pairwise F_{ST} were then averaged at the level of the population as a measure of how differentiated a population was from others relative to the expected differentiation based on IBD (Table S1).

Method S2: Drift load study (Study 2)

Plant growth in greenhouse conditions

Seeds of different maternal plants (hereafter seed families) were collected in late summer 2020 and 2021 in natural populations and stored in separate bags at 4°C under dark and dry conditions. We sowed up to five seeds for each seed family in each population in each of two pots filled with a 3:1 mixture of potting mix (LM-111 All Purpose Mix, Lambert, Rivière-Ouelle QC, Canada) and turface. Pots were randomly distributed across 128-cell propagation trays. Germination occurred in growth chambers under 12h light, 21°C day, 14°C night, 60% RH for four weeks. Pots were watered daily for a week, then every other day. Trays were randomized within growth chambers twice a week. At the end of the germination, seedlings were randomly thinned to one per pot and plants were fertilized once (13 mg/L, Jack's Professional 15-5-15 + CaMg LX Fertilizer, JR Peters Inc., Allentown PA). Seedlings were then vernalized to simulate winter (4°C, low light for 12h per day) for six weeks, which is required for most populations to initiate reproduction (Perrier et al., 2025).

After vernalization, one replicate per seed family was retained and moved to a greenhouse with daylength extended to 16h with artificial light (600 µmol/m2/s) to induce flowering. Initial temperature of 23°C day / 19°C night (+/- 5°C) was increased after two months to daytime temperature of 25°C and plants were shaded to simulate a light environment of an open forest. These temperatures reflected average summer temperatures across the range (Table S4). Plants were watered as needed. Fertilizer was applied at 1000ppm (N) every two days for two months, then one to two times a week. Plants were treated with fungicide (two times before and two times after vernalization, Banrot 40 WP, Everris NA Inc., Dublin, OH; 0.9 g/L) to avoid root rot. During flowering, plants were sprayed with pesticide once a week to reduce damage by thrips (neem oil and liquid soap 1:1, diluted in water at 13ml/L, and spinosad diluted in water at 16.5ml/L).

Generation of within- and between- population crosses

Within-population crosses (WPC) were conducted between random combinations of plants in each population, with each plant serving as pollen recipient (mother) twice and as pollen donor twice. For between-population crosses (BPC), we created15 lines (Table S2B) by assembling populations at roughly similar latitudes into pairs to minimize the effects of divergent local adaptation. Of the 19 populations available, 11 served as the maternal population in one pair, and the paternal

population in the other pair, while the remaining 8 contributed to only one pair, serving only as a either maternal or a paternal population (Fig. 2, Table S2B). Pollinations were conducted between random combinations of maternal and paternal plants from designated populations.

All crosses were performed by hand on emasculated flowers with pollen collected from the selected donor. Three replicate crosses for each parental combination were conducted for WPC and two for BPC. Mature fruits were collected and stored in individual envelopes at 4°C under dry and dark conditions.

Raising of WPC and BPC offspring

We grew 18-25 individuals for each WPC and 18-20 for BPC, with each seed family represented twice but occasionally once or three times, depending on seed availability (Table S2). For each pot, we sowed two seeds and thinned to one individual before vernalization. Plants were raised as described above, except that germination lasted five weeks and vernalization lasted seven weeks. This experiment was stopped once all plants that successfully bolted had flowered for five weeks

Recording of fitness traits

We tracked fitness across the life cycle. We scored germination and survival of each seedling once a week before simulated winter. After plants were moved to the greenhouse, survival and flowering (opening of at least one flower) were scored three times a week for each plant. Once a plant started flowering, we recorded its reproductive output as the sum of five weekly counts of open flowers.

Method S3: Local adaptation study (Study 3)

Raising of plants in the common garden

Plants of the 23 populations selected for the local adaptation study were raised in three common gardens in two cohorts (Method S3 Figure A, B). We used greenhouse-reared seeds (WPC, Method S2) for 20 populations, and field collected seeds for the remaining three (Table S3). For each population, we selected 15 seed families (randomly duplicated if fewer available) to be used in the experiment. With few exceptions (1 family out of 345 in the first cohort, 3 out of 347 in the second), we used the same seed families in both cohorts.

We raised the summer cohort from fall 2022 through late summer 2023 (Perrier et al., 2025). For each garden, two seeds per family (three for field seeds, one if few seeds) were sowed in each of two pots, thinned to one per pot after germination. Seeds were germinated for 6 weeks and vernalized for 7 weeks as described in Method S2. In spring 2023, seedlings were acclimated outdoors for 1 week (Charlottesville, VA, USA), then transplanted into each common garden, starting with the southernmost garden to reflect the earlier start of the growing season at lower latitudes. The transplant date (Method S3 Table A) was chosen as the date when mean daily temperature was consistently above 10°C in spring (estimated using data for 2018–2022 from the PRISM database, https://prism.oregonstate.edu, accessed 13/05/2024, more details in Perrier et al. 2025). Sowing dates were planned to ensure seedlings were transplanted at the same life stage across common gardens.

The winter cohort was initiated in fall 2023 and followed through late spring 2024. The seed sowing design was similar to the summer cohort. Seeds were germinated for 5 weeks as in Method S2, then seedlings were acclimated for four days to the colder outdoor conditions of late fall (Charlottesville, VA), except for the southernmost garden where seedlings were kept in indoors growing conditions that better reflected the temperatures at the garden's location. Seedlings were then transplanted in each garden starting with the northernmost garden, reflecting the earlier start of winter. Transplant date (Method S3 Table B) was chosen as the date two weeks before the first day where mean daily temperature was below 4°C in fall, or two weeks before the first day of frost, depending on which occurred first (dates estimated using PRISM data for 2018–2022). Sowing dates were planned accordingly.

In both cohorts, plants were transplanted into 30 blocks of 23 individuals (Method S3 Figure B), with one individual per population per block resulting in 30 replicates of a population

in each garden (final replication in Table S3, more details in Perrier et al., 2025). The distribution of populations within blocks, and of seed families for each population between blocks, was determined though randomization, and was changed between both cohorts. The same garden locations were used for both cohorts.

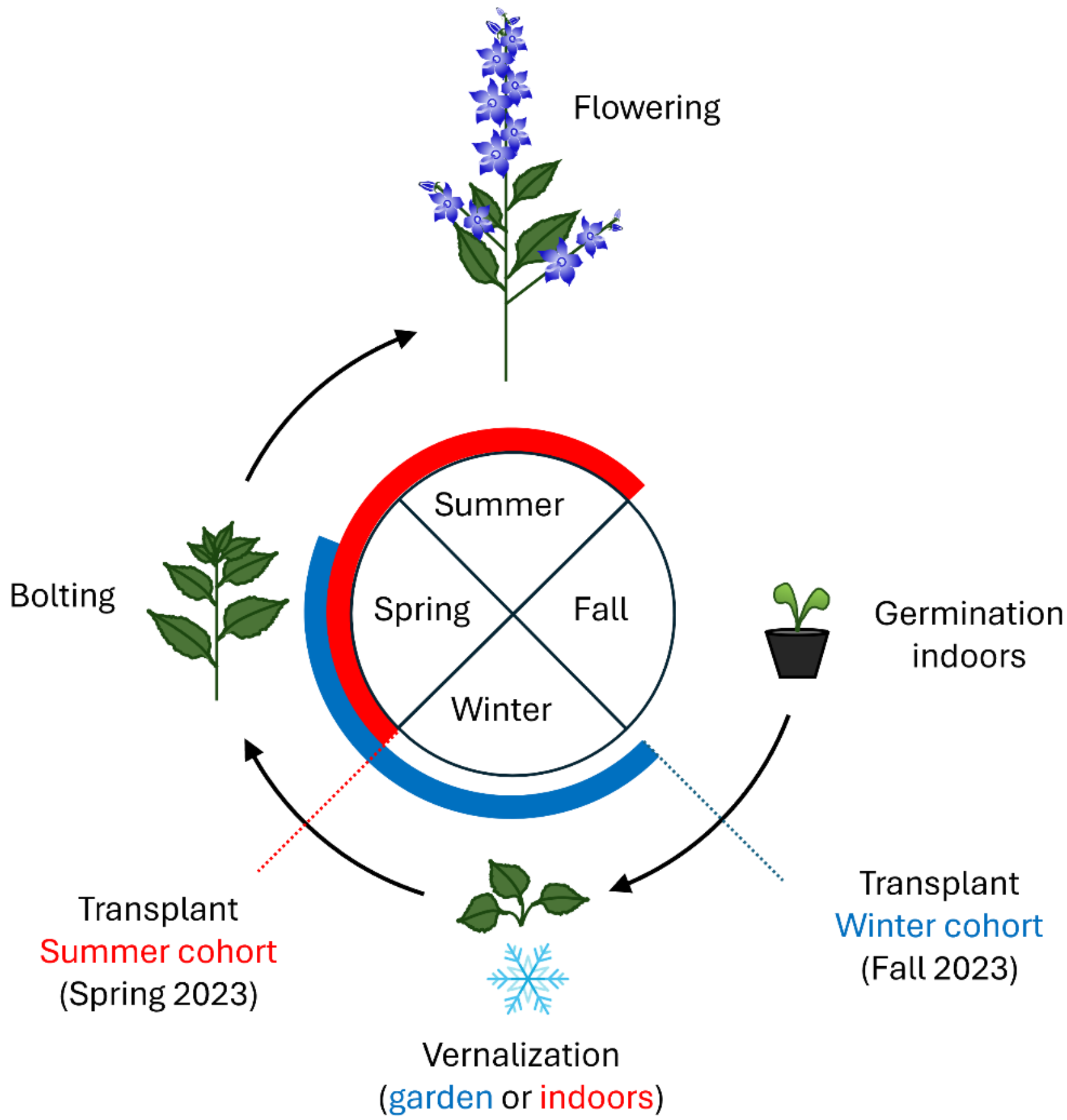

Method S3 Figure A: Life cycle of *C. americana* split between winter and summer cohorts. The common garden experiment consisted of a summer cohort (red, spring and summer 2023) and a winter cohort (blue, fall 2023 – spring 2024). Plants were germinated indoors for both cohorts, then transplanted into each garden before winter for the winter cohort, and after a simulated winter indoors for the summer cohort. Together, the two cohorts allowed determining the effects of climate across most of the species' life cycle.

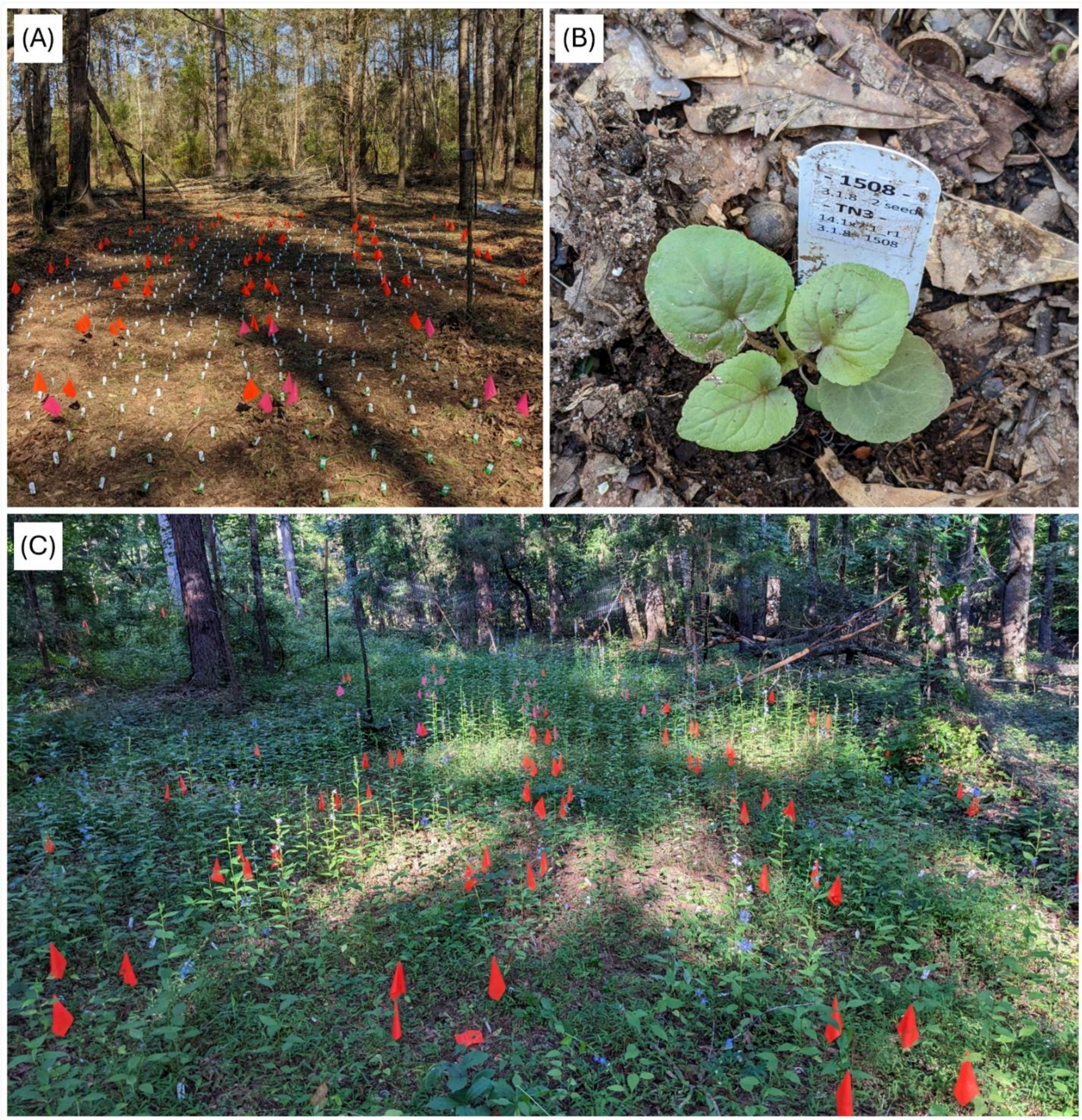

Method S3 Figure B: Pictures of the common garden CG-Int (Clemson, SC), summer cohort (A,B) Garden right after transplant. **(C)** Garden on the first visit in summer.

Method S3 Table A: Location of common gardens (CG) and schedule of the summer cohort

	Latitude	Longitude	Transplant	Visit 1	Visit 2	Visit 3
CG-Core	38.08079	-84.47134	08/04/2023	16/07/2023	12/08/2023	09/09/2023
CG-Int	34.68746	-82.87249	22/03/2023	14/07/2023	13/08/2023	06/09/2023
CG-Rear	30.45723	-84.33319	02/03/2023	12/07/2023	17/08/2023	-

For CG-Rear, 98% of plants were considered mature at the second visit, thus all plants were collected, and the garden was not visited a third time.

Method S3 Table B: Schedule of the winter cohort

	Transplant	Visit 1	Visit 2
CG-Core	29/10/2023	28/03/2024	05/06/2024
CG-Int	12/11/2023	25/03/2024	31/05/2024
CG-Rear	14/11/2023	26/03/2024	02/06/2024

Recording of fitness traits

We defined five traits representing fitness components at different life stages. *Survival over winter* (binary) was the survival of plants in the winter cohort from transplant in fall (2023) until the first recording date in early spring (2024). *Survival until reproduction* (binary) was the survival of plants in the summer cohort from transplant in early spring (2023) until the end of the cohort in summer. *Bolting* (binary) was the initiation of bolting of winter-cohort plants and was assessed over two visits (2024). *Flowering* (binary) was the initiation of flowering of summer-cohort plants (2023) that had bolted. *Reproductive output* was the sum of fruits, flowers and buds (see below) of flowering plants in the summer cohort.

Estimation of reproductive output

In the summer cohort, mature plants were collected at each visit to determine reproductive output. Plants were considered mature if they had been flowering for at least 5 weeks (first fruits starting to turn brown, see Perrier et al., 2025). This timing was chosen to allow plants sufficient time to produce the bulk of their flowers and buds, but at the same time to prevent mature fruits from opening and dispersing seeds. At the southern garden (CG-Rear), all plants were collected at the second visit as 95% of plants had flowered (Perrier et al., 2025), and of these 98% were considered mature. In the other two gardens, all reproductive plants were collected on the third visit, in September, regardless of maturation. In most natural populations, flowering is finished by September (personal obs.), and open flowers at this time are unlikely to produce mature fruits before the onset of winter. Stopping the experiment then thus allows for an accurate representation of the contribution of phenology to reproductive success, an important component of adaptive divergence between populations across the range (Perrier et al., 2025). To collect, plants were cut at the base of the flowering stalk, stored in individual bags and dried. We scored reproductive output as the sum of the number of fruits, flowers and buds on each plant. We also measured the stem diameter at the base of the flowering stalk.

For 325 plants (~20% of all plants that reproduced, Method S3 Table C), reproductive output could not be scored accurately. These included plants that matured before collection started and lost fruits, plants where the main stem was broken, and 15 plants that did not reach fruit maturation in the southern garden. We predicted reproductive output for these based on the relationship between reproductive output and stem diameter. We estimated this relationship across

gardens for each population using linear models with reproductive output as the dependent variable (assuming normal distribution), and the squared stem diameter (in mm) as fixed effect (average R^2 = 0.60, Method S3 Table C). In cases where the predicted reproductive output was lower than the minimum observed reproductive output for that population, the predicted value was assigned to be the minimum observed value.

Method S3 Table C: Summary statistics for estimated reproductive output

Population	$N_{measured}$	$N_{predicted}$	R^2	P
FL83	49	15	0.52	2.76E-09
FL81	31	27	0.30	8.13E-04
AL2	53	14	0.77	5.62E-18
MS6	55	17	0.76	1.88E-18
AL23	53	16	0.45	1.85E-08
AL22	52	14	0.74	1.16E-16
AL79	58	11	0.74	2.23E-18
AL21	45	18	0.62	8.67E-11
MS8	59	6	0.71	3.54E-17
GA1	46	15	0.43	5.39E-07
ALBG	55	12	0.58	1.01E-11
TN3	65	9	0.58	1.04E-13
TN34	61	15	0.51	5.41E-11
AR2	57	11	0.67	4.75E-15
KY5	54	11	0.68	7.36E-15
KY1	40	11	0.47	5.60E-07
MO2	52	14	0.71	2.25E-15
OH1	60	8	0.52	5.62E-11
KS60	62	11	0.61	5.86E-14
IN5	55	10	0.59	3.52E-12
OH119	57	6	0.63	8.07E-14
IN4	53	17	0.62	2.18E-12
IA19	43	15	0.57	5.38E-09

Summary statistics include the replication (*N*) of individuals with measures of reproductive output used to predict this trait for individuals without measures, the coefficient of determination (R^2) and the significance (*P*) of the relationship between reproductive output and stem diameter for each population, across gardens.

Climate variables

We calculated 14 climate variables that captured variation in temperature and precipitation at key life stages. For each common garden, daily mean, minimum and maximum temperatures (°C) were recorded during the experiment with dataloggers (temperature), and nearby weather stations (precipitation, <50km away, https://www.ncdc.noaa.gov, accessed 05/06/2024). For each population, we obtained daily temperature and precipitation data between 2018 and June 2023 from the PRISM using the *prism* R package (Edmund & Bell, 2015), based on the populations' coordinates.

We defined three time periods (hereafter "seasons", Method S3 Table D) to capture the effect of climate on key life stages in the local adaptation experiment. *Winter,* experienced by the winter cohort, was the time between the date of acclimation (or transplant for CG-Rear) and the first visit in early spring, capturing survival over winter and cueing of reproduction (i.e. vernalization). *Spring,* experienced by the summer cohort, was the time between the date of acclimation until first flowering, capturing vegetative growth and the development of the flowering stalk and buds. *Summer,* also experienced by the summer cohort, was the time between first flowering and the end of the experiment capturing reproduction. These time periods were calculated for each common garden using the dates in Method S3 Table D. Seasons were calculated for each population for each of the five years before the start of the experiment (2018 – 2022, for winter calculated from fall of each year to spring of year + 1), then averaged across years.

For each season we calculated averages of daily mean, minimum and maximum temperature, and the sum of daily precipitation. We also calculated the number of days of frost and of vernalization (Method S3 Table E). Finally, we calculated five bioclimatic variables representing seasonal variation (Method S3 Table E) using the function *biovars* of the R package *dismo* (Hijmans et al., 2024) based on monthly averages of daily minimum and maximum temperatures, and monthly sum of daily precipitation. For gardens, we calculated monthly averages using a one-year daily dataset, combining temperature (from dataloggers) and precipitation (from weather station) recorded during the seasons defined in Method S3 Table D, complemented by temperature and precipitation data obtained from weather stations to bridge gaps. For populations, we obtained daily temperatures and precipitation from PRISM to calculate monthly averages for each year between 2018 and 2022, which were then averaged across years.

Parametrization of the hierarchical mixed-effect model

Models were parametrized using the R packages *lme4* (Bates et al., 2015) and *blme* (Dorie et al., 2026) and were optimized using the *Nelder_Mead* optimizer.

*Model = blmer(fitness ~ poly(ΔPC1, degree = 2) * site + (1 | population), data = data, control = lmerControl(optimizer = "Nelder_Mead", optCtrl = list(maxfun = 100000)))*

Method S3 Table D: Schedule for seasonal climate variables

Season	Start	End
Winter 2023-2024	*Acclimation*	*First visit in spring*
CG-Core	25/10/2023	28/03/2024
CG-Int	08/11/2023	25/03/2024
CG-Rear	14/11/2023	26/03/2024
Populations	05/11/2023	26/03/2024
Spring 2023	*Transplant*	*First flower*
CG-Core	08/04/2023	07/06/2023
CG-Int	22/03/2023	19/05/2023
CG-Rear	01/03/2023	09/05/2023
Populations	22/03/2023	22/05/2023
Summer 2023	*First flower*	*Last visit*
CG-Core	07/06/2023	07/09/2023
CG-Int	19/05/2023	04/09/2023
CG-Rear	09/05/2023	14/08/2023
Populations	22/05/2023	29/08/2023

First flower date was the earliest date within a garden at which plants of the summer cohort opened their first flowers. The date of opening of the first flower was inferred for each plant based on the development of the flowering stalk (as in Perrier et al., 2025). Dates for populations were calculated as the average date across the common gardens.

Method S3 Table E: Description of climatic variables

Variables	Description
Seasonal climatic variables	
Tmean	Seasonal average of daily mean temperatures
Tmin	Seasonal average of daily minimum temperatures
Tmax	Seasonal average of daily maximum temperatures
PPTsum	Seasonal sum of daily precipitation
DayFrost	Number of days during winter where Tmin < 0°C
DayVerna	Number of days during winter where Tmean < 4°C
Bioclimatic variables	
Bio 2	Mean diurnal range (mean of monthly (max. temp. – min. temp.))
Bio 3	Isothermality ((Bio 2 / Bio 7) ×100)
Bio 4	Temp. seasonality (standard deviation of monthly mean temp. ×100)
Bio 7	Temp. annual range (max. temp. of warmest month – min. temp. of coldest month)
Bio 15	Precipitation seasonality (coefficient of variation of monthly total precipitation)

Tmean, Tmin, Tmax and PPTsum were calculated for each season (winter, spring, summer), DayFrost and DayVerna only for winter.

Supplementary Tables

Table S1: Population genetics study (Study 1) – Populations used and summary statistics

Pop.	Latitude [°N]	Longitude [°E]	Sequenced individuals	Θ_π (× 10^3)	Θ_W (× 10^3)	Differentiation (F_{ST} ~ geography)
FL83	30.56468	-84.95982	9	6.8089	6.5403	0.0933
FL81	30.81165	-85.22535	10	6.7192	6.6644	0.1704
AL2	31.54792	-87.51453	10	7.4527	7.7212	0.0127
MS6	31.99803	-89.35603	10	6.3271	6.3735	0.0994
AL23	32.19395	-86.78484	10	7.4147	7.8436	0.0109
AL8	32.25610	-86.49330	8	7.1251	7.0367	0.0670
AL22	32.50376	-87.50489	9	7.3975	7.3661	0.0248
AL19	32.60699	-88.19192	10	6.8132	7.0820	0.0794
AL79	32.92932	-88.20820	8	7.3443	7.5878	-0.0168
AL21	33.49109	-86.79829	9	7.4240	7.2680	0.0211
MS9	33.92778	-89.01710	10	6.0470	5.8701	0.1431
MS8	34.40427	-88.83093	10	7.2869	7.3836	-0.0100
GA1	34.60074	-84.69664	10	7.2319	7.2683	0.0085
ALBG	34.65346	-86.51638	7	6.9523	6.6850	0.0664
TN3	35.30932	-90.06770	9	7.3577	7.9155	-0.0533
TN34	36.08222	-86.29611	10	7.7173	8.7418	-0.0719
AR2	36.15235	-94.30464	9	6.1749	6.2712	0.0374
KY5	37.36005	-84.77186	9	7.5310	9.2381	-0.1045
KY1	38.09215	-84.98688	10	7.5346	8.5197	-0.0703
MO2	38.83025	-92.28509	10	7.4832	8.7379	-0.0990
KS60	39.04742	-95.68152	9	7.2858	7.7920	-0.0768
IN5	39.14583	-86.54833	9	6.7762	7.0198	0.0031
IN7	39.87011	-86.16020	10	7.5731	8.4827	-0.0948
OH119	39.88500	-83.99700	9	7.3589	7.7707	-0.0536
IL10	40.61750	-89.01778	10	7.0531	7.5470	-0.0500
IN2	41.01731	-85.23872	10	6.7843	6.9255	-0.0167
IA12	41.69417	-93.67417	8	7.2769	7.8771	-0.0929
IA17	42.46194	-90.63611	10	7.0473	7.6022	-0.0531
MI2	42.62369	-85.43706	10	5.7276	5.9761	0.1070
MN8	44.02861	-92.43222	10	6.4514	6.8711	-0.0055
MN117	44.90100	-93.19200	10	6.9598	7.3902	-0.0753

Differentiation represents the population-average residuals of the relationship between linearized pairwise F_{ST} estimates and pairwise geographic distances (Method S1).

Table S2A: Drift load study (Study 2) – Populations used, sampling for parental and within-population cross (WPC) seed families, and population-level heterosis

Population	Latitude [°N]	Longitude [°W]	Seed families (parental)	Seed families (WPC)	Individuals (WPC)	Lifetime Fitness (WPC)	Drift load (average heterosis)	F_{ST} (population-average)
FL83	30.56468	-84.95982	17	14	20	203.34	-	-
FL81	30.81165	-85.22535	16	13	19	175.58	-	-
**AL2**	31.54793	-87.51453	17	15	25	210.16	-0.0590	0.3640
**MS6**	31.99803	-89.35603	17	14	25	123.78	0.0842	0.4495
**AL23**	32.19395	-86.78484	14	14	23	160.78	0.0350	0.3578
AL8	32.25610	-86.49330	18	15	20	155.60	-	-
AL22	32.50376	-87.50489	13	8	20	135.40	-	-
**AL79**	32.92932	-88.20820	13	14	24	106.15	0.0669	0.3260
MS9	33.92778	-89.01710	11	11	20	132.67	-	-
MS8	34.40427	-88.83093	12	11	20	101.26	-	-
**GA1**	34.60074	-84.69664	11	12	28	144.63	0.0389	0.3531
**ALBG**	34.65346	-86.51638	14	13	23	139.51	0.2409	0.4037
**TN3**	35.30932	-90.06770	11	13	25	117.46	0.1072	0.2854
**TN34**	36.08222	-86.29611	15	12	24	153.21	0.0618	0.2659
**AR2**	36.15235	-94.30465	16	15	25	125.02	0.1555	0.3927
**KS60**	39.04742	-95.68152	17	14	25	96.90	0.1578	0.2888
**IN5**	39.14583	-86.54833	16	14	24	153.83	0.1894	0.3447
OH119	39.88500	-83.99700	12	10	18	124.56	-	-
IN4	40.44806	-86.93444	15	12	20	121.38	-	-
**PA27 ***	41.00800	-80.08326	25	19	20	-	0.2150	0.2821
**OH64 ***	41.11500	-81.51810	25	18	19	-	0.1410	0.2987
**MI127 ***	41.92300	-86.58336	25	14	14	-	0.3355	0.3333
**IA10 ***	42.07300	-93.67250	25	21	21	-	0.1965	0.6393
**MI126 ***	42.32100	-85.34209	25	19	19	-	0.1820	0.3699
**WI128 ***	43.15000	-90.04498	25	19	20	-	0.1280	0.5152
**MN117 ***	44.90100	-93.19248	25	17	17	-	0.2340	0.3089
**MN118 ***	45.02600	-95.88795	25	20	21	-	0.3150	0.3699

Populations used to estimate drift load are indicated in bold, the other populations contributed to a single BPC (Table S2B). For eight populations (*), drift load was calculated from heterosis data in Koski *et al.* (2019), and F_{ST} was calculated from data in Koski *et al.* (2022); lifetime fitness not available. Drift load was calculated as the average heterosis (based on lifetime fitness) across a population's two BPC lines (Table S2B).

Table S2B: Drift load study (Study 2) – Population pairs, sampling and heterosis estimates

Mother population	Father population	Seed families (BPC)	Individuals (BPC)	Lifetime fitness (BPC)	Heterosis
FL83	MS6	14	20	182.91	0.1183
FL81	AL2	13	20	187.78	-0.0264
AL2	AL8	15	20	166.13	-0.0916
MS6	AL23	14	20	149.43	0.0502
AL23	MS9	14	20	149.64	0.0199
AL79	GA1	14	19	126.91	0.0121
MS8	ALBG	13	20	163.74	0.3601
GA1	AL22	8	19	149.22	0.0658
ALBG	AL79	13	20	137.78	0.1217
TN3	TN34	11	20	137.17	0.0136
TN34	AR2	13	20	154.43	0.1100
AR2	TN3	15	20	145.60	0.2009
KS60	IN4	13	20	123.74	0.1338
IN5	KS60	14	20	148.16	0.1818
OH119	IN5	10	18	166.62	0.1970
PA27 *	IA10 *	20	20	-	0.1230
OH64 *	MN118 *	17	18	-	0.3230
MI127 *	MN117 *	14	14	-	0.5090
IA10 *	MI126 *	20	21	-	0.2700
MI126 *	WI128 *	17	17	-	0.0940
WI128 *	MI127 *	19	21	-	0.1620
MN117 *	OH64 *	17	18	-	-0.0410
MN118 *	PA27 *	20	22	-	0.3070

Heterosis was calculated based on lifetime fitness of within and between population crosses. For eight populations (*), heterosis data from Koski *et al.* (2019), lifetime fitness not available.

Table S3: Local adaptation study (Study 3) – Populations, sampling, and population-level lifetime fitness in common gardens (CG)

				Summer cohort (2023)						Winter cohort (2023 – 2024)								
				Families			Individuals			Families			Individuals			Lifetime fitness		
ID	Latitude [° N]	Longitude [° E]	Seed type	CG-Center	CG-Int	CG-Rear	CG-Center	CG-Int	CG-Rear	CG-Center	CG-Int	CG-Rear	CG-Center	CG-Int	CG-Rear	CG-Center	CG-Int	CG-Rear
FL83	30.56468	-84.95982	Crosses	13	15	15	20	29	30	15	15	15	29	28	26	0.89	1.20	1.43
FL81	30.81165	-85.22535	Crosses	14	15	15	20	30	29	15	15	15	29	30	30	0.32	0.83	0.66
AL2	31.54793	-87.51453	Crosses	15	15	15	20	30	30	14	15	15	27	28	28	0.76	1.32	1.55
MS6	31.99803	-89.35603	Crosses	13	15	15	20	30	30	15	15	15	28	30	29	1.35	1.42	1.80
AL23	32.19395	-86.78484	Crosses	15	15	15	20	30	29	15	15	15	29	29	29	1.02	1.28	1.75
AL22	32.50376	-87.50489	Crosses	13	15	15	20	30	29	15	15	15	29	29	29	1.03	1.04	1.60
AL79	32.92932	-88.20820	Crosses	13	15	15	19	30	30	15	15	13	29	28	25	1.00	1.27	1.36
AL21	33.49109	-86.79829	Crosses	13	15	15	20	30	29	15	15	15	29	28	28	0.78	1.42	0.59
MS8	34.40427	-88.83093	Crosses	13	15	14	20	29	29	15	15	15	30	30	30	1.23	1.12	0.83
GA1	34.60074	-84.69664	Crosses	14	15	15	19	30	30	15	14	14	28	26	25	0.56	1.46	0.31
ALBG	34.65346	-86.51638	Crosses	13	15	15	19	30	29	13	15	15	25	29	30	1.08	1.13	0.38
TN3	35.30932	-90.06770	Crosses	13	15	15	20	30	29	15	15	15	29	29	30	1.37	1.41	0.81
TN34	36.08222	-86.29611	Crosses	15	15	15	20	30	30	15	15	15	30	30	30	1.27	1.05	0.84
AR2	36.15235	-94.30465	Crosses	13	15	15	20	29	30	15	15	15	29	30	30	1.24	1.00	0.50
KY5	37.36005	-84.77186	Field	14	15	15	20	29	30	15	15	15	29	28	30	0.99	1.29	0.12
KY1	38.09215	-84.98688	Field	11	15	15	19	29	30	13	14	12	26	26	22	1.06	1.29	0.00
MO2	38.83025	-92.28509	Field	12	14	14	18	27	29	14	14	13	27	26	26	1.18	0.92	0.00
OH1	39.03935	-84.32774	Field	12	15	15	20	29	27	15	15	15	30	30	30	1.53	1.37	0.00
KS60	39.04742	-95.68152	Crosses	14	15	15	20	30	29	15	15	15	30	30	28	1.27	1.11	0.11
IN5	39.14583	-86.54833	Crosses	13	15	15	19	30	29	15	15	14	29	30	26	1.30	1.13	0.00
OH119	39.88500	-83.99700	Crosses	13	15	15	20	30	29	15	15	15	28	29	27	1.40	1.31	0.48
IN4	40.44806	-86.93444	Crosses	13	15	15	19	30	29	15	15	15	29	29	29	1.42	1.36	0.11
IA19	41.56611	-90.47500	Crosses	13	15	15	19	29	30	14	15	14	27	30	28	1.14	0.88	0.00

Families and individuals depict the final number of unique seed families and individuals per population that were transplanted in each garden and tracked for fitness. Only pots with successful and timely germination were transplanted (96% of 4140 pots). In CG-Center in the summer cohort, the first 10 blocks had to be later excluded due to a mudslide. In total, 3768 seedlings were transplanted and tracked over the two cohorts. Seed-family level lifetime fitness was log10 transformed before calculating population averages.

Table S4 Local adaptation study (Study 3) – Climate variables and the first PC axis (PC1) for populations and common gardens (CG)

	Winter						Spring				Summer									
	Tmean	Tmin	Tmax	PPTsum	DayFrost	DayVerna	Tmean	Tmin	Tmax	PPTsum	Tmean	Tmin	Tmax	PPTsum	bio2	bio3	bio4	bio7	bio15	PC1
Populations																				
FL83	13.93	7.63	20.22	524.69	15.00	3.00	21.69	15.06	28.31	154.39	27.47	22.04	32.90	542.59	12.03	41.56	628.11	28.94	28.03	5.83
FL81	13.83	7.69	19.97	471.12	12.80	3.00	21.70	15.42	27.98	180.03	27.59	22.40	32.77	521.06	11.68	40.79	636.11	28.62	26.86	5.76
AL2	12.05	5.26	18.84	484.74	36.40	9.00	20.01	12.70	27.33	177.67	26.63	20.99	32.28	458.20	12.99	42.40	675.34	30.64	26.79	4.22
MS6	11.22	4.80	17.63	670.41	36.40	12.20	19.57	12.85	26.29	263.80	26.63	21.05	32.21	415.68	12.41	39.60	712.46	31.35	30.49	3.78
AL23	11.30	4.92	17.68	577.98	36.20	11.60	19.54	12.72	26.37	156.19	26.64	21.12	32.17	391.93	12.30	39.59	708.14	31.07	14.81	3.54
AL22	10.74	4.34	17.14	627.51	40.60	16.40	19.35	12.63	26.07	191.60	26.56	20.91	32.21	435.92	12.47	39.46	731.88	31.61	22.74	3.40
AL79	10.30	4.12	16.48	743.63	39.80	17.40	19.18	12.73	25.63	230.98	26.57	21.07	32.07	410.90	12.04	37.90	749.24	31.77	24.23	3.19
AL21	10.45	5.32	15.59	757.26	30.20	18.00	19.27	13.65	24.88	220.04	26.34	21.55	31.13	443.60	10.32	34.34	740.00	30.07	25.82	2.94
MS8	9.03	3.23	14.83	751.16	44.20	26.60	18.96	12.93	25.00	205.60	26.75	21.39	32.12	366.89	11.59	35.04	817.35	33.08	30.54	2.21
GA1	8.28	2.45	14.11	646.62	51.20	28.20	17.52	10.89	24.16	146.76	25.24	19.61	30.86	333.53	11.99	36.93	773.44	32.48	24.77	0.95
ALBG	7.98	2.43	13.52	803.97	53.40	34.00	17.36	11.28	23.43	205.16	25.05	19.78	30.33	375.49	11.37	35.25	787.57	32.26	28.87	0.86
TN3	7.67	2.60	12.73	675.94	44.80	34.40	18.46	13.02	23.91	193.33	26.59	21.58	31.59	274.90	10.60	31.77	871.38	33.37	34.51	0.84
TN34	6.84	0.49	13.20	706.96	69.40	41.80	16.24	9.11	23.38	189.93	25.01	18.70	31.32	400.29	13.18	37.70	829.34	34.97	26.60	0.26
AR2	5.37	-0.46	11.21	408.07	75.20	55.20	16.01	10.29	21.73	277.09	24.88	19.47	30.29	304.75	11.55	32.66	896.39	35.37	39.55	-1.45
KY5	4.65	-0.76	10.05	639.24	76.40	62.20	14.93	8.74	21.12	186.46	23.50	17.89	29.11	424.26	11.56	33.74	858.56	34.26	20.69	-1.84
KY1	3.97	-1.19	9.12	518.98	80.20	68.20	14.69	8.90	20.48	157.17	23.58	18.15	29.01	387.16	11.06	31.68	895.37	34.90	20.17	-2.66
MO2	2.64	-2.75	8.03	331.60	87.60	73.60	15.09	9.32	20.85	178.42	24.70	19.08	30.33	315.03	11.44	30.22	1006.40	37.85	35.34	-3.28
OH1	3.18	-1.77	8.13	441.33	86.00	74.80	14.25	8.44	20.06	179.55	23.43	18.02	28.84	384.76	10.78	30.80	922.27	35.00	22.41	-3.29
KS60	2.07	-3.97	8.11	194.38	103.80	80.00	14.91	8.93	20.89	181.55	25.09	19.55	30.62	336.89	12.02	30.58	1042.56	39.32	61.91	-3.79
IN5	2.49	-2.47	7.45	494.78	93.00	81.00	13.94	7.99	19.89	158.25	23.34	17.76	28.92	380.28	11.05	30.59	956.30	36.13	21.21	-3.76
OH119	1.74	-3.10	6.57	402.92	96.60	88.20	13.30	7.63	18.96	160.35	22.94	17.49	28.40	323.92	10.73	29.88	965.83	35.90	22.42	-4.67
IN4	0.45	-4.09	5.00	315.31	102.20	96.60	12.86	7.17	18.55	150.08	22.73	17.07	28.38	261.93	10.73	28.96	1019.50	37.06	27.80	-5.78
IA19	-1.56	-6.11	2.99	245.64	113.60	106.40	12.02	6.58	17.46	163.73	22.87	17.68	28.05	271.78	10.19	25.56	1113.19	39.86	40.63	-7.27
Common gardens																				
CG-Rear	13.63	8.48	20.51	698.13	4.00	2.00	19.23	13.91	25.32	180.33	25.68	21.87	30.61	464.22	11.04	38.38	588.13	28.76	45.95	4.35
CG-Int	8.50	0.68	16.07	707.66	79.00	27.00	16.68	10.77	24.12	285.21	22.94	18.76	28.67	493.39	12.71	39.25	711.61	32.37	57.53	0.72
CG-Center	6.46	0.91	14.15	514.40	69.00	53.00	16.42	10.33	22.94	122.99	22.14	18.32	26.77	392.85	11.95	37.67	716.13	31.72	47.85	-1.16

Variable description and calculation detailed in Method S3

Table S5 Local adaptation study (Study 3) – Summary of the principal component analysis (PCA) on seasonal and bioclimatic variables

		PC1	PC2	PC3	PC4
Variance explained (%)		76.65	8.81	5.86	4.59
Cumulative variance (%)		76.65	85.46	91.32	95.9
Loadings					
Winter	Tmean	**0.26**	-0.02	0.00	0.02
	Tmin	**0.26**	-0.05	-0.06	0.09
	Tmax	**0.26**	0.00	0.05	-0.04
	PPTsum	0.16	**-0.22**	**-0.51**	**-0.31**
	DayFrost	**-0.26**	0.04	0.10	-0.13
	DayVerna	**-0.26**	0.00	0.04	0.03
Spring	Tmean	**0.26**	0.08	0.00	0.12
	Tmin	0.25	0.11	-0.10	0.24
	Tmax	**0.26**	0.05	0.08	0.02
	PPTsum	0.08	**0.40**	**-0.54**	**-0.49**
Summer	Tmean	0.25	**0.23**	0.02	0.13
	Tmin	0.24	**0.24**	-0.08	**0.25**
	Tmax	0.25	0.21	0.12	0.01
	PPTsum	0.20	-0.21	**0.24**	0.02
Bioclim.	Bio 2 (Mean diurnal range)	0.15	0.08	**0.50**	**-0.64**
	Bio 3 (Isothermality)	0.25	-0.08	**0.23**	-0.22
	Bio 4 (Temperature seasonality)	-0.25	0.16	-0.01	0.05
	Bio 7 (Temperature annual range)	-0.25	0.20	0.11	-0.11
	Bio 15 (Precipitation seasonality	0.08	**0.70**	0.14	0.10

Data only shown for principal components (PC) 1 to 4, as the cumulative contribution of the remaining PCs explained less than 5% of the variance. Loadings highlighted in bold indicate the main contributing variables to a given PC axis (upper quartile based on absolute loading for each PC).

Table S6: Model comparisons between testing linear and quadratic effects of latitude

Dependent variable	*N*	$AICc_{linear}$	$AICc_{square}$	R^2_{linear}	R^2_{square}
Study 1 - Population genetics					
Θ_W	31	-490.82	**-494.00**	0.00	0.14
Θ_π	31	-521.84	-522.88	-0.02	**0.06**
Differentiation	31	-78.82	**-83.95**	0.30	0.44
Study 2 - Drift load					
Heterosis	19	**-43.33**	-40.51	0.54	0.53

The best model (bold) was identified by the lowest AICc value. For models with similar AICc ($|\Delta AICc|<2$), the best model was the one with highest R^2 (bold).

Table S7A: Local adaptation study (Study 3) – Estimates of the effect of ΔPC1 on relative lifetime fitness

	β ΔPC1	*β* $ΔPC1^2$
CG-Center	0.024	-0.003
CG-Int	0.003	-0.003
CG-Rear	-0.103	0.003

Values depict the estimated slope coefficients (*β*) of the linear and quadratic effect of ΔPC1 in each common garden (CG).

Table S7B: Local adaptation study (Study 3) – Comparison of the effect of ΔPC1 on relative lifetime fitness between common gardens (CG)

	β Common gardens * ΔPC1	
CG-Center vs CG-Int	0.02	
CG-Center vs CG-Rear	**0.11**	**
CG-Int vs CG-Rear	**0.09**	***

Values depict the estimated pairwise contrast (*β*) of the effect of ΔPC1between common gardens (based on the garden - ΔPC1 interaction) obtained from Tukey's tests. Estimates with *P*-values < 0.05 in bold; ** $P<0.01$, *** $P<0.001$.

Table S8A: Local adaptation study (Study 3) – Test of variation in lifetime fitness across common gardens and population latitude.

Fixed effects	X^2	
Common garden (CG)	**87.92**	***
Latitude	**9.36**	**
CG * latitude	**136.88**	***

The dependent variable was population-average lifetime fitness, assumed to follow Gaussian distributions. All fixed effects were tested in one model, optimized with the bobyqa optimizer to improve convergence. Test statistics include the chi-squared value of the model, bold if P-values < 0.05; ** P<0.01, *** P<0.001. Marginal R^2_m = 0.70 (fixed effect only); R^2_c = 0.83 (fixed and random effects). Results for random effects are not shown.

Table S8B: Local adaptation study (Study 3) – Comparison of lifetime fitness between common gardens (CG)

	β Common gardens	
CG-Center vs CG-Int	-0.11	
CG-Center vs CG-Rear	**0.58**	***
CG-Int vs CG-Rear	**0.69**	***

Values depict the estimated pairwise differences (β) between common gardens obtained from Tukey's tests. Estimates with P-values < 0.05 in bold; *** P<0.001.

Table S8C: Local adaptation study (Study 3) – Estimates of the effect of latitude on lifetime fitness

	β Latitude	β Latitude^2
CG-Center	0.419	-0.005
CG-Int	0.364	-0.005
CG-Rear	-0.780	0.009

Values depict the estimated slope coefficients (β) of the linear and quadratic effect of latitude in each common garden (CG).

Table S8D: Local adaptation study (Study 3) – Comparison of the effect of ΔPC1 on relative lifetime fitness between common gardens (CG)

	β Common gardens * Latitude	
CG-Center vs CG-Int	**0.06**	**
CG-Center vs CG-Rear	**0.21**	***
CG-Int vs CG-Rear	**0.15**	***

Values depict the estimated pairwise contrast (β) of the effect of latitude between common gardens (based on the garden – latitude interaction) obtained from Tukey's tests. Estimates with P-values < 0.05 in bold; ** P<0.01, *** P<0.001.

Supplementary Figures

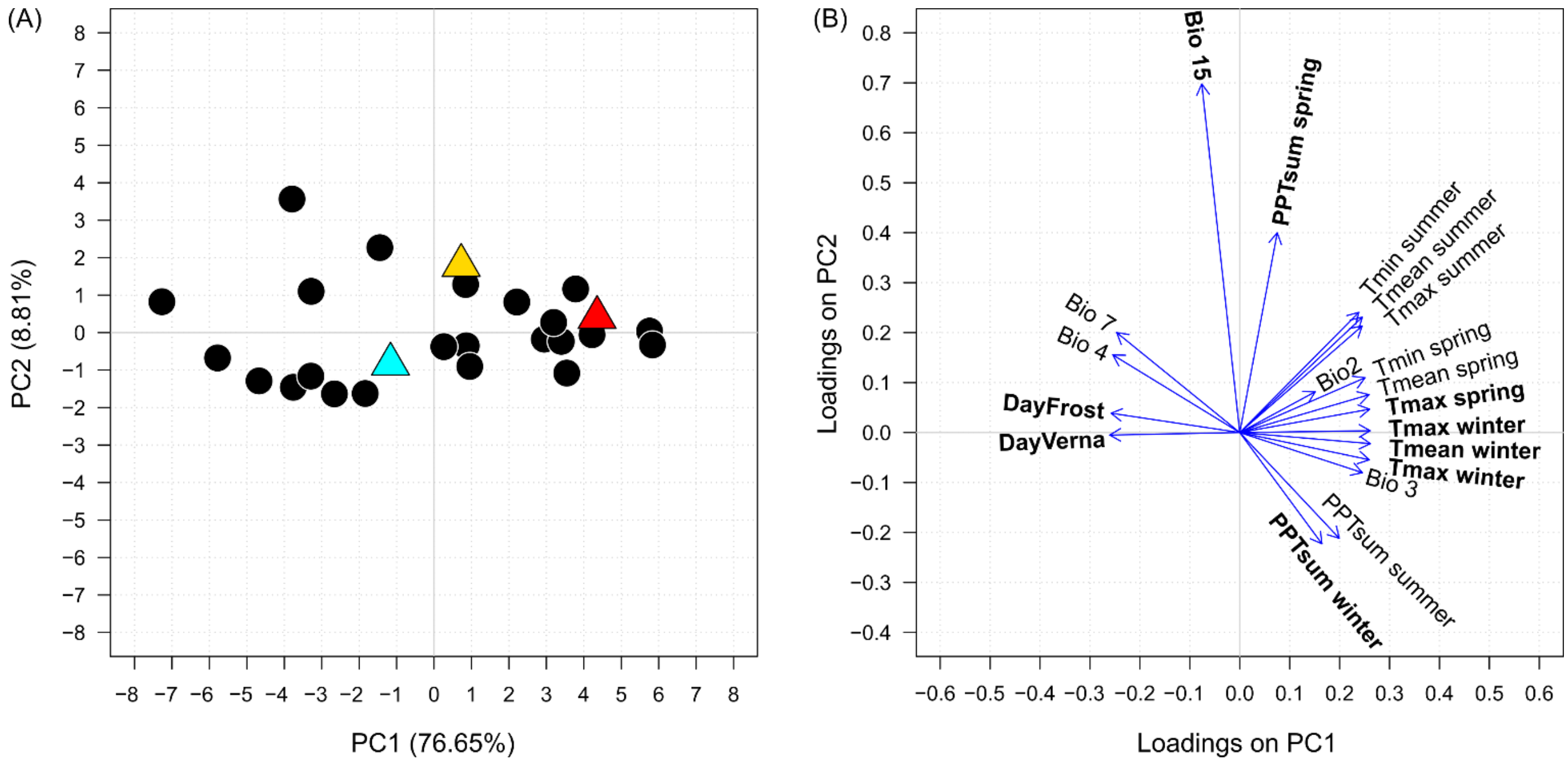

Figure S1. Local adaptation study (Study 3) – Estimation of climate differences between gardens and populations

(A) Environmental variation across the 23 populations (dots) included in the experiment is represented by their relative position along the first two axes of a principal component analysis, based on 19 seasonal and bioclimatic variables estimated for each populations' location of origin. Common gardens (triangles; cyan, CG-Center; yellow, CG-Int; red, CG-Rear) were projected onto the populations' PC space based on the same climate variables. **(B)** The contribution of seasonal and bioclimate variables to the first two principal components are indicated by arrows, with the main contributing variables labeled in bold (Table S5).

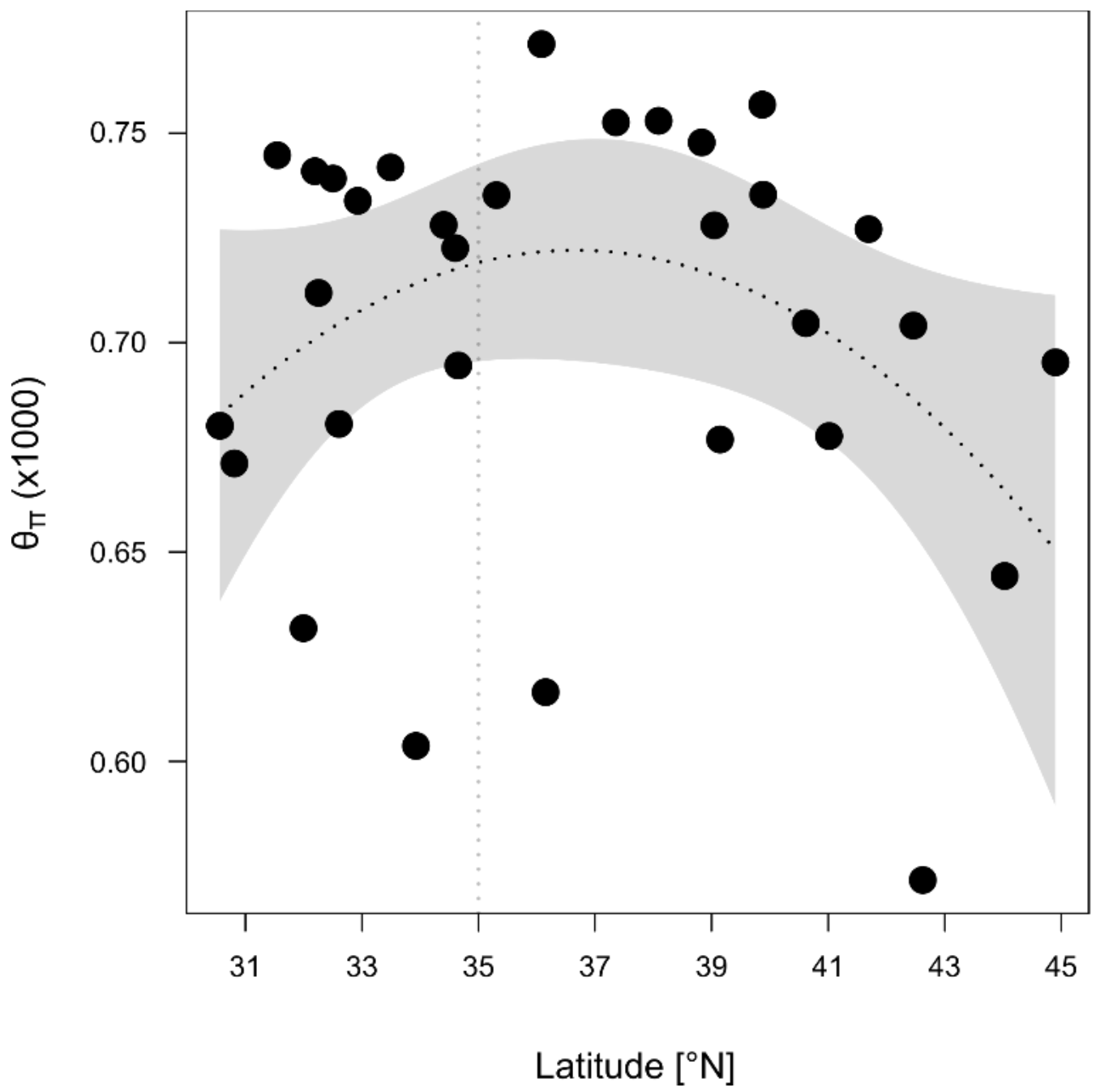

Figure S2: **Population genetic study (Study 1) – Variation in genetic diversity across latitudes**
Genetic diversity within population based on Tajimas' θ_π estimated for each population (dots). Vertical dotted line represents the separation between the rear edge (<35°N) and the expanded range. Dashed black line represents the model-predicted relationship between the estimate and population latitude, with the 95% confidence interval indicated as shading. Test statistics reported in Table 1.

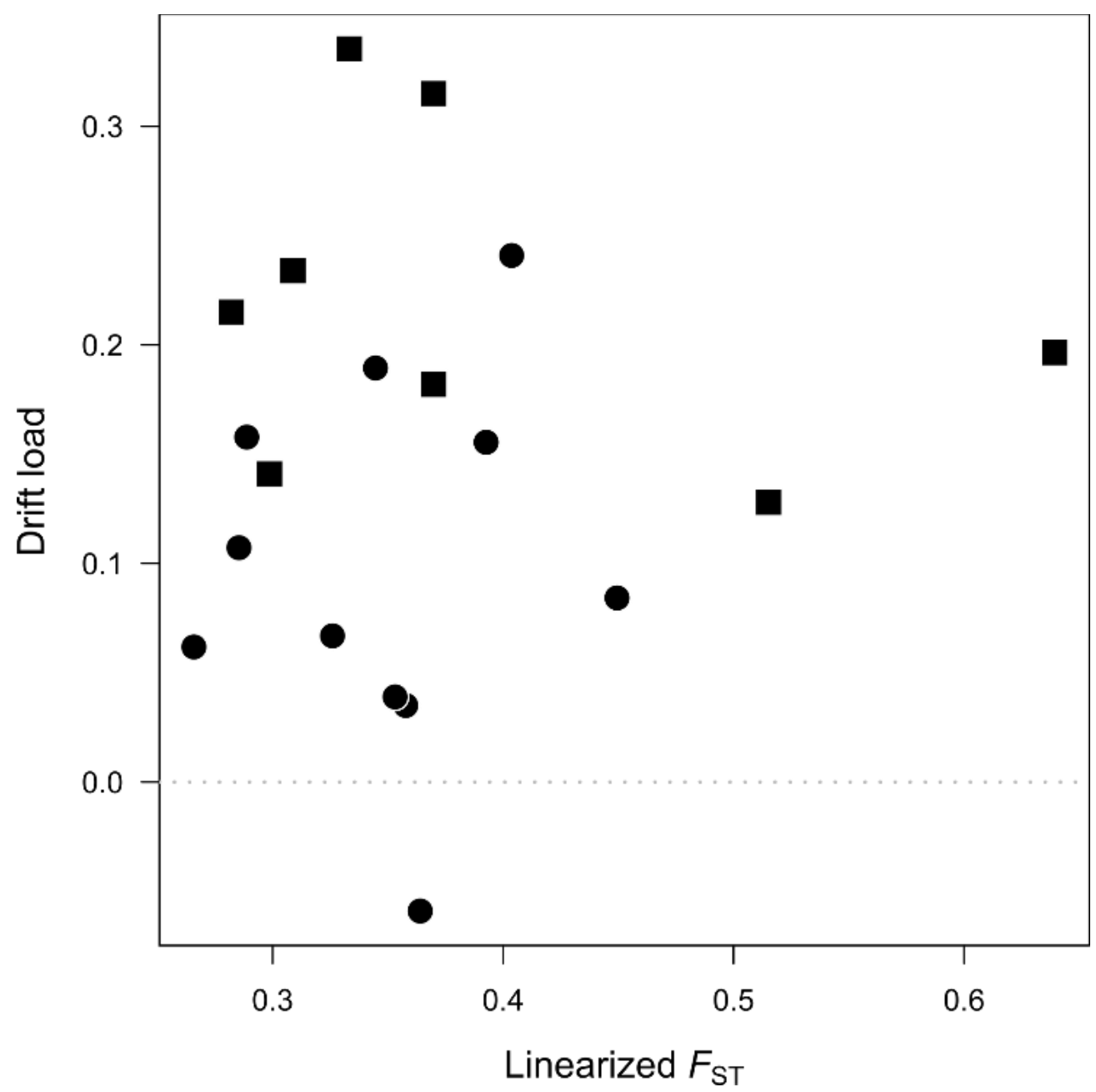

Figure S3: Drift load study (Study 2) – Variation in the expression of drift load with population differentiation (F_{ST})

Drift load of each population (dots) including additional populations from Koski et al. (squares) plotted again linearized F_{ST}. The lack of significant relationship indicates that drift load is not associated with genetic differentiation among populations.

References

Andrews, K. R., Good, J. M., Miller, M. R., Luikart, G., & Hohenlohe, P. A. (2016). Harnessing the power of RADseq for ecological and evolutionary genomics. *Nature Reviews Genetics*, *17*(2), 81–92. https://doi.org/10.1038/nrg.2015.28

Andrews, S. (2010). FastQC: A quality control tool for high throughput sequence data. https://www.bioinformatics.babraham.ac.uk/projects/fastqc/

Bates, D., M. Mächler, B. Bolker, & Walker, S. 2015. Fitting linear mixed-effects models using lme4. Journal of Statistical Software 67:1–48. https://doi.org/10.18637/jss.v067.i01

Baird, N. A., Etter, P. D., Atwood, T. S., Currey, M. C., Shiver, A. L., Lewis, Z. A., Selker, E. U., Cresko, W. A., & Johnson, E. A. (2008). Rapid SNP discovery and genetic mapping using sequenced RAD Markers. *PLOS ONE*, *3*(10), e3376. https://doi.org/10.1371/journal.pone.0003376

Danecek, P., Auton, A., Abecasis, G., Albers, C. A., Banks, E., DePristo, M. A., Handsaker, R. E., Lunter, G., Marth, G. T., Sherry, S. T., McVean, G., Durbin, R., & 1000 Genomes Project Analysis Group. (2011). The variant call format and VCFtools. *Bioinformatics*, *27*(15), 2156–2158. https://doi.org/10.1093/bioinformatics/btr330

Danecek, P., Bonfield, J. K., Liddle, J., Marshall, J., Ohan, V., Pollard, M. O., Whitwham, A., Keane, T., McCarthy, S. A., Davies, R. M., & Li, H. (2021). Twelve years of SAMtools and BCFtools. *GigaScience*, *10*(2), giab008. https://doi.org/10.1093/gigascience/giab008

Dorie, V., Bates, D., M. Mächler, B. Bolker, & Walker, S. (2026), blme: Bayesian Linear Mixed-Effects Models. R package version 1.0-7. Available at: http://CRAN.R-project.org/package=blme (accessed 02 March 2026).

Eckert, C. G., Samis, K. E., & Lougheed, S. C. (2008). Genetic variation across species' geographical ranges: The central–marginal hypothesis and beyond. *Molecular Ecology*, *17*(5), 1170–1188. https://doi.org/10.1111/j.1365-294X.2007.03659.x

Edmund, H., & Bell, K. (2015). prism: Download data from the Oregon prism project. *Website.* https://cran.r-project.org/package=prism

Hijmans, R. J., Phillips, S., Leathwick, J., & Elith, J. (2024). dismo: Species Distribution Modeling. *Website.* https://cran.r-project.org/web/packages/dismo/index.html

Karney, C. F. F. (2013). Algorithms for geodesics. *Journal of Geodesy*, *87*(1), 43–55. https://doi.org/10.1007/s00190-012-0578-z

Koski, M. H., Galloway, L. F., & Busch, J. W. (2022). Hybrid breakdown is elevated near the historical cores of a species' range. *Proceedings of the Royal Society B: Biological Sciences*, *289*(1971), 20220070. https://doi.org/10.1098/rspb.2022.0070

Koski, M. H., Layman, N. C., Prior, C. J., Busch, J. W., & Galloway, L. F. (2019). Selfing ability and drift load evolve with range expansion. *Evolution Letters*, *3*(5), 500–512. https://doi.org/10.1002/evl3.136

Li, H. (2013). Aligning sequence reads, clone sequences and assembly contigs with BWA-MEM. *arXiv*. https://doi.org/10.48550/arXiv.1303.3997

Li, H. (2014). Toward better understanding of artifacts in variant calling from high-coverage samples. *Bioinformatics*, *30*(20), 2843–2851. https://doi.org/10.1093/bioinformatics/btu356

Li, H., & Durbin, R. (2009). Fast and accurate short read alignment with Burrows–Wheeler transform. *Bioinformatics*, *25*(14), 1754–1760. https://doi.org/10.1093/bioinformatics/btp324

Pembleton, L. W., Cogan, N. O. I., & Forster, J. W. (2013). StAMPP: An R package for calculation of genetic differentiation and structure of mixed-ploidy level populations. *Molecular Ecology Resources*, *13*(5), 946–952. https://doi.org/10.1111/1755-0998.12129

Perrier, A., Turner, M. C., & Galloway, L. F. (2025). Shifts in vernalization and phenology at the rear edge hold insight into the adaptation of temperate plants to future milder winters. *New Phytologist*, *246*(3), 1377–1389. https://doi.org/10.1111/nph.70005

Prior, C. J., Layman, N. C., Koski, M. H., Galloway, L. F., & Busch, J. W. (2020). Westward range expansion from middle latitudes explains the Mississippi River discontinuity in a forest herb of eastern North America. *Molecular Ecology*, *29*(22), 4473–4486. https://doi.org/10.1111/mec.15650

R Core Team. (2025). R: a language and environment for statistical computing. R Foundation for Statistical Computing.

Rochette, N. C., Rivera-Colón, A. G., & Catchen, J. M. (2019). Stacks 2: Analytical methods for paired-end sequencing improve RADseq-based population genomics. *Molecular Ecology*, *28*(21), 4737–4754. https://doi.org/10.1111/mec.15253

Rousset, F. (1997). Genetic differentiation and estimation of gene flow from F-statistics under isolation by distance. *Genetics*, *145*(4), 1219–1228. https://doi.org/10.1093/genetics/145.4.1219

Slatkin, M. (1993). Isolation by distance in equilibrium and non-equilibrium populations. *Evolution*, *47*(1), 264–279. https://doi.org/10.2307/2410134

Weir, B. S., & Cockerham, C. C. (1984). Estimating F-Statistics for the analysis of population structure. *Evolution*, *38*(6), 1358. https://doi.org/10.2307/2408641
